# Genome-wide methylation data improves dissection of the effect of smoking on body mass index

**DOI:** 10.1371/journal.pgen.1009750

**Published:** 2021-09-09

**Authors:** Carmen Amador, Yanni Zeng, Michael Barber, Rosie M. Walker, Archie Campbell, Andrew M. McIntosh, Kathryn L. Evans, David J. Porteous, Caroline Hayward, James F. Wilson, Pau Navarro, Chris S. Haley

**Affiliations:** 1 MRC Human Genetics Unit, Institute of Genetics and Cancer, University of Edinburgh, Edinburgh, United Kingdom; 2 Faculty of Forensic Medicine, Zhongshan School of Medicine, Sun Yat-Sen University, China; 3 Centre for Genomic and Experimental Medicine, Institute of Genetics and Cancer, University of Edinburgh, Edinburgh, United Kingdom; 4 Centre for Clinical Brain Sciences, Chancellor’s Building, 49 Little France Crescent, Edinburgh BioQuarter, Edinburgh, United Kingdom; 5 Division of Psychiatry, University of Edinburgh, Edinburgh, United Kingdom; 6 Centre for Global Health Research, Usher Institute, University of Edinburgh, Edinburgh, United Kingdom; 7 Roslin Institute and Royal (Dick) School of Veterinary Studies, University of Edinburgh, Edinburgh, United Kingdom; Newcastle University, UNITED KINGDOM

## Abstract

Variation in obesity-related traits has a genetic basis with heritabilities between 40 and 70%. While the global obesity pandemic is usually associated with environmental changes related to lifestyle and socioeconomic changes, most genetic studies do not include all relevant environmental covariates, so the genetic contribution to variation in obesity-related traits cannot be accurately assessed. Some studies have described interactions between a few individual genes linked to obesity and environmental variables but there is no agreement on their total contribution to differences between individuals. Here we compared self-reported smoking data and a methylation-based proxy to explore the effect of smoking and genome-by-smoking interactions on obesity related traits from a genome-wide perspective to estimate the amount of variance they explain. Our results indicate that exploiting omic measures can improve models for complex traits such as obesity and can be used as a substitute for, or jointly with, environmental records to better understand causes of disease.

## Author summary

Most diseases and health-related outcomes are influenced by genetic and environmental variation. Hundreds of genetic variants associated with obesity-related traits, like body mass index (BMI), have been previously identified, as well as lifestyles contributing to obesity risk. Furthermore, certain combinations of genetic variants and lifestyles may change the risk of obesity more than expected from their individual effects. One obstacle to further research is the difficulty in measuring relevant environmental impacts on individuals. Here, we studied how genetics (genome-wide markers) and tobacco smoking (self-reported) affect BMI. We also used DNA methylation, a blood-based biomarker, as a proxy for to self-reported information to assess tobacco usage. We incorporated the effect of interactions between genetics and self-reported smoking or methylation. We estimated that genetics accounted for 50% of the variation in BMI. Self-reported smoking status contributed only 2% of BMI variation, increasing to 22% when estimated using DNA methylation. Interactions between genes and smoking contributed an extra 10%. This work highlights the potential of using biomarkers to proxy lifestyle measures and expand our knowledge on disease and suggests that the environment may have long-term effects on our health through its impact on the methylation of disease-associated loci.

## Introduction

Variation in obesity-related traits such as body mass index (BMI) has a complex basis with heritabilities ranging from 40 to 70%, with the genetic variants detected to date explaining up to 5% of BMI variation [1]. In addition to genetics, studies suggest that the increase in obesity prevalence in recent decades is linked to environmental causes, such as dietary changes and a more sedentary lifestyle [2–5]. The fact that all relevant environmental effects have not been accounted for in genetic studies has potentially reduced GWAS power to detect susceptibility variants. On top of this, several studies suggest that gene-by-environment interactions also play an important role in obesity and other complex traits [2,6–10] and many researchers are focusing on finding interactions between specific genes and certain environments. Genotype-by-age interactions and genotype-by-sex interactions have also been detected for several health-related traits [10–12]. Recently, when performing GWAS on traits like BMI, lipids, and blood pressure, several studies have stratified their samples on the basis of smoking status or have explicitly modelled interactions leading to identification of new genetic variants associated with those traits [13–15]. Some studies have attempted to quantify the overall contribution of genetic interactions with smoking. Robinson, et al. [12] estimated them to explain around 4% of BMI variation in a subset of unrelated UK Biobank samples. In contrast, also in UK Biobank, using a new approach that only requires summary statistics, Shin & Lee [16] estimated the contributions of the interactions to be much smaller: 0.6% of BMI variation.

In this study, we aim to estimate the contribution of smoking and its interaction with genetic variation to obesity variation, using self-reported measures of smoking and a methylomic proxy of smoking exposure. We hypothesised that use of a proxy, rather than self-reported smoking, and fitting genome-by-smoking interactions would lead to more a more accurate model. DNA methylation is an epigenetic mark that can be affected by genetics and environmental exposures [17–22]. Variation in methylation is correlated with gene expression, plays a crucial role in development, in maintaining genomic stability [23–25], and has been associated with disease [26–30] and aging [31,32]. Epigenome-wide association analyses (EWAS) have identified multiple associations between DNA methylation levels at specific genomic locations and smoking [18,33–35]. These so-called *signatures* of smoking in the epigenome can help discriminate the smoking status of the individuals in a cohort [19], and, if sufficiently accurate, could be an improvement on self-reported measures, by adding information not captured (accurately) in the self-reported measure, such as passive smoking or real quantity of tobacco smoked.

Here, we aim to estimate the contribution to obesity variation of smoking and its interaction with genetic variation in two different cohorts, using self-reported measures of smoking and a methylomic proxy for smoking. Thus, we measured the contribution of smoking-associated methylation signatures and genome-by-methylation interactions to trait variation. We performed analyses in both sexes jointly and independently and also including genome-by-smoking-by-sex interactions, and we showed that omics data can be exploited as proxies for environmental exposures to improve our understanding of complex trait architecture. We observed that using an appropriate set of CpG sites, methylation can be used to model trait variation associated with smoking, and genome-by-smoking interactions suggesting potential applications for better prediction and prognosis of complex disease and expanding these modelling approaches to other environments and traits.

## Results

**Fig 1:**
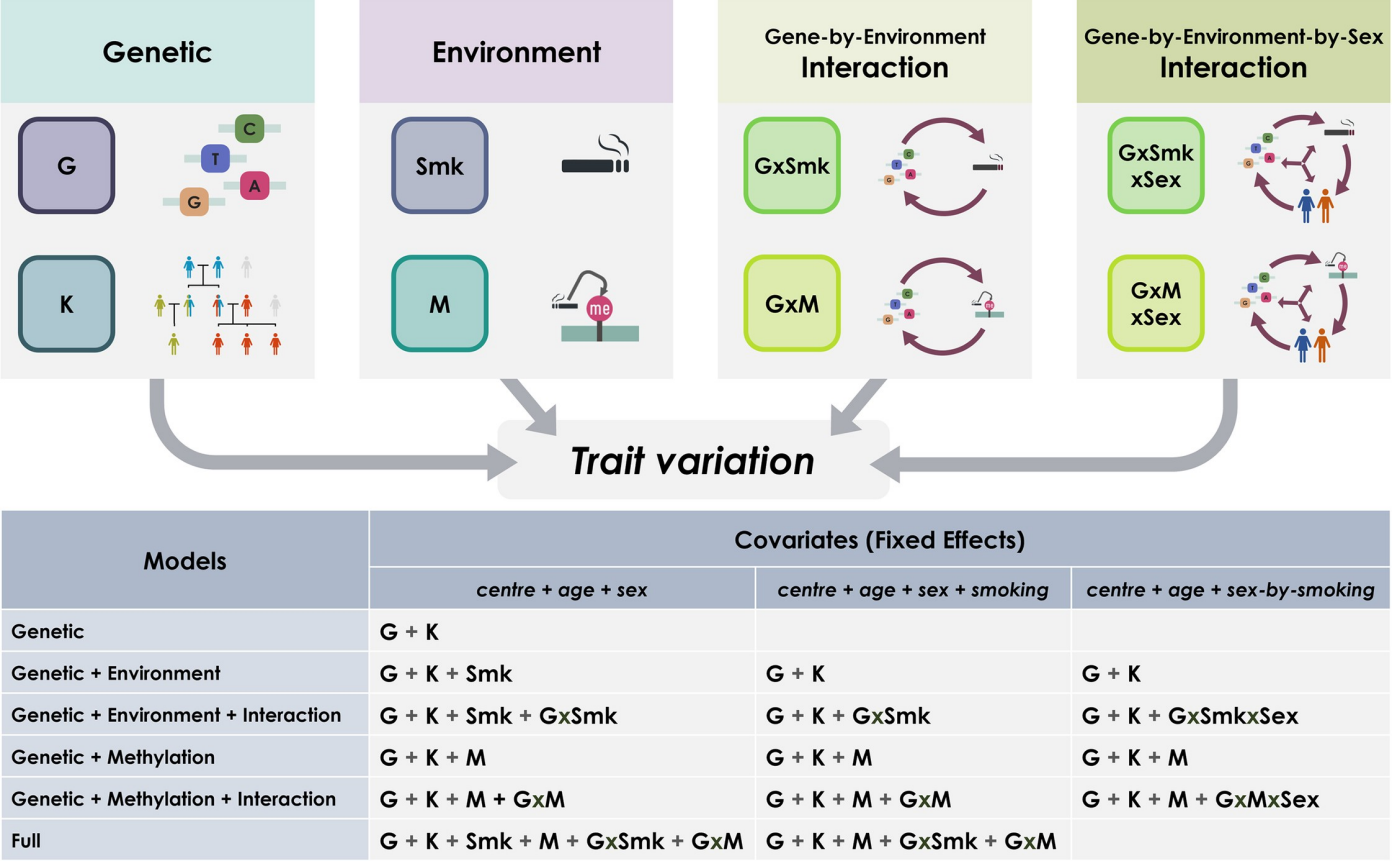
Summary of the experimental design of the study. The panels (above) represent the genetic and environmental components contributing to trait variation and used in the models (table below). Each cell shows the included random effects in each combination of model (row) and fixed effects (columns). G: Genomic, K: Kinship, GxSmk: Genome-by-Smoking, M: Methylation, GxM: Genome-by-Methylation, GxSmkxSex: Genome-by-Smoking-by-Sex, GxMxSex: Genome-by-Methylation-by-Sex. Models applied to different data sets varied depending on data availability.

The aim of this work was to explore the influence of smoking and genome-by-smoking interactions on trait variation, modelling them from self-reported information and using DNA methylation in both sexes jointly and separately. We used a variance component approach to fit a linear mixed model including a set of covariance matrices representing: two genetic effects (G: common SNP-associated genetic effects and K: pedigree-associated genetic effects not captured by the genotyped markers at a population level; the inclusion of matrix K in the analyses allows to use the related individuals in the sample), environmental effects reflecting impact of smoking (modelled as fixed or random effects), and genome-by-smoking effects (GxSmk) representing sharing of both genetics (G) and environment (smoking, Smk), and we estimated the proportion of variation that each component explained for seven obesity-related measures: weight, body mass index (BMI), waist circumference (waist), hip circumference (hips), waist-to-hip ratio (WHR), fat percentage (fat%), and HDL cholesterol (HDL) as well as height, to serve as a negative control. We defined the environment using either self-reported questionnaire data or its associated methylation signature as a proxy. A summary of the experimental design used in this study is shown in Fig 1. For more detailed information, see Methods.

### Self-reported smoking status

#### Generation Scotland

**Fig 2:**
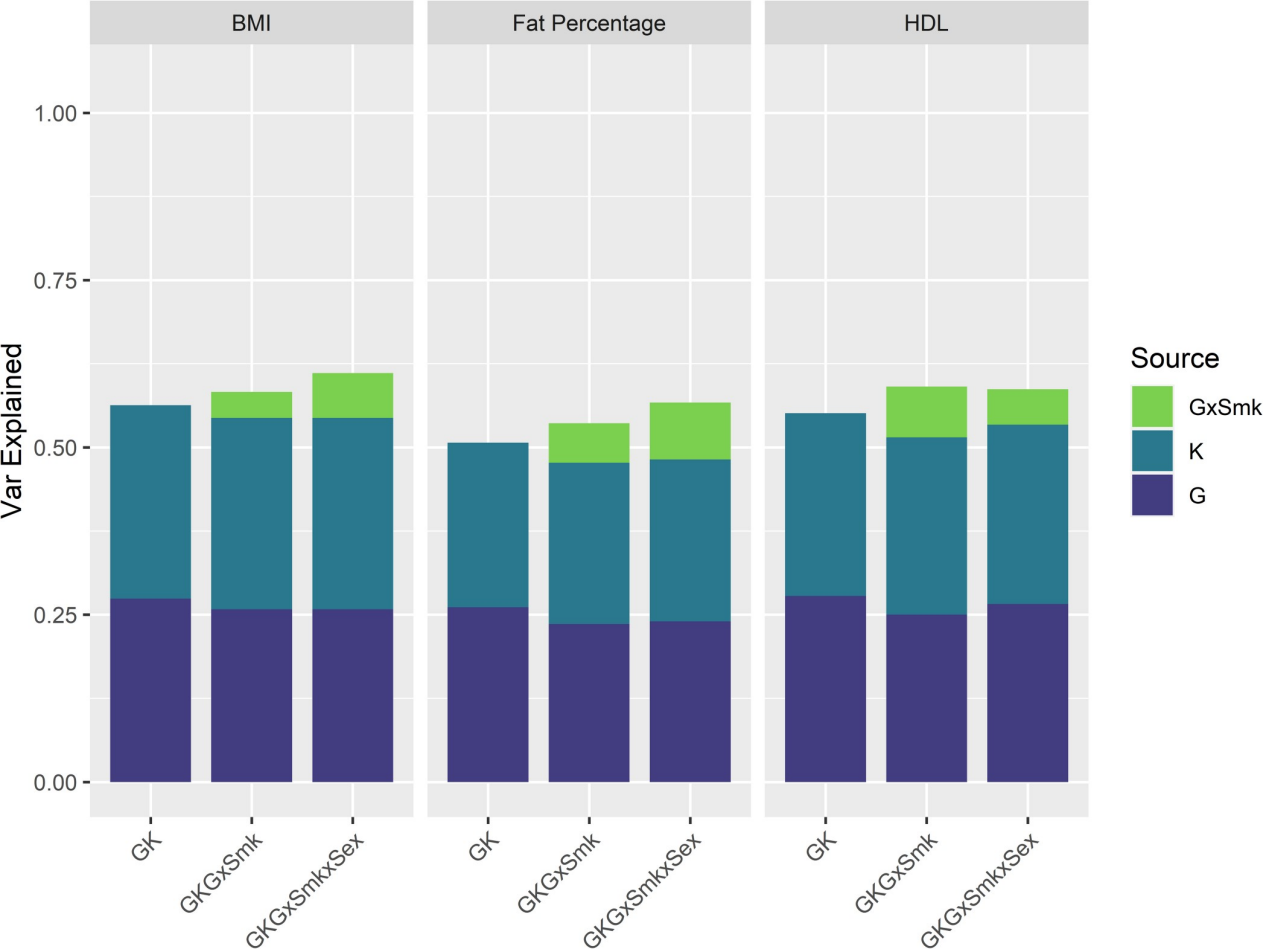
Proportion of trait variation explained by genetic and interaction sources in GS18K. Proportion of BMI, fat percentage, and HDL variance (y-axis) explained by each of the genetic and interaction sources in the corresponding models (x-axis). G: Genomic, K: Kinship, GxSmk: Genome-by-Smoking, GxSmkxSex: Genome-by-Smoking-by-Sex.

Fig 2 shows the estimates of the proportion of BMI, fat percentage, and HDL variance explained by different sources included in the linear mixed models in ~18K individuals in Generation Scotland (GS18K). Results for other traits are displayed in Table 1, S1 Fig, and full details of the analyses for all traits including estimates, standard errors, and log-likelihood ratio tests (LRT) are shown in S1 Table.

**Table 1:** Summary of interaction results for all cohorts. Results of GKGxSmk model for all traits in GS18K and meta-analysis of the recruitment centre-based sub-cohorts in UK Biobank. The table shows, for each trait, the proportion of the phenotypic variance explained (Var), its standard error (SE), the log-likelihood ratio test P value (LRT P, only for the interaction), the meta-analysis P value (P), for each of the components in the model: Genetic (G), Kinship (K) and Genome-by-Smoking interaction (GxSmk). Highlighted P values indicate nominally significant results for the GxSmk component.

Trait	Source	GS18K			UKB Meta Analysis		
		Var	SE	LRT P	Var	SE	P
**Height**	**G**	0.483	0.022		0.629	0.009	
**Height**	**K**	0.429	0.024		0.328	0.006	
**Height**	**GxSmk**	0.012	0.014	0.2041	0.001	0.003	0.7640
**Weight**	**G**	0.270	0.024		0.355	0.007	
**Weight**	**K**	0.302	0.027		0.242	0.018	
**Weight**	**GxSmk**	0.049	0.021	**0.0098**	0.022	0.008	**0.0050**
**BMI**	**G**	0.258	0.024		0.318	0.008	
**BMI**	**K**	0.286	0.028		0.236	0.021	
**BMI**	**GxSmk**	0.039	0.021	**0.0336**	0.025	0.007	**0.0009**
**Waist**	**G**	0.181	0.024		0.261	0.004	
**Waist**	**K**	0.313	0.028		0.214	0.021	
**Waist**	**GxSmk**	0.023	0.022	0.1534	0.017	0.007	**0.0119**
**Hips**	**G**	0.212	0.024		0.296	0.009	
**Hips**	**K**	0.271	0.028		0.179	0.028	
**Hips**	**GxSmk**	0.027	0.023	0.1185	0.020	0.007	**0.0048**
**WHR**	**G**	0.130	0.023		0.217	0.005	
**WHR**	**K**	0.198	0.027		0.151	0.013	
**WHR**	**GxSmk**	0.019	0.023	0.2011	0.012	0.006	**0.0437**
**Fat%**	**G**	0.236	0.025		0.301	0.006	
**Fat%**	**K**	0.241	0.028		0.224	0.013	
**Fat%**	**GxSmk**	0.059	0.023	**0.0036**	0.021	0.005	**0.0000**
**HDL**	**G**	0.250	0.024		NA	NA	
**HDL**	**K**	0.265	0.027		NA	NA	
**HDL**	**GxSmk**	0.076	0.022	**0.0002**	NA	NA	

The heritability estimates of all analysed traits (i.e., proportion of the variance captured by G and K matrices together) are consistent with previous estimates in the same cohort [36]. The estimated contributions of smoking status (and the other covariates) to trait variation ranged between 0.35% (for height, assessed as a negative control, as we do not expect to find the same type of effects as with obesity-related measures) and 1.2% (for HDL cholesterol) and are shown in S2 Table. When included as random effect, smoking explained between 0.1% (for height) and 2.5% (for HDL cholesterol) of trait variation (S1 Table). Our models identified significant genome-by-smoking interactions for weight, BMI, fat percentage and HDL cholesterol (with log-likelihood ratio tests showing that the models including the interaction were significantly better), explaining between 4 and 8% of trait variation (Table 1), similar to the values of Robinson et al. [12] for BMI. When the interactions included sex (genome-by-smoking-by-sex interactions) the component was significant for all traits, and explained variance ranging between 2–9% (S2 Table).

#### UK Biobank

We sought to replicate the results observed in Generation Scotland with data from the UK Biobank cohort (UKB). Analyses were run in four sub-cohorts for computational reasons (G1, G2, G3 and G4, grouping individuals in geographically close recruitment centres; for more information see Methods and S3 Table), with the two sexes considered jointly and separately in three different analyses (the sample size of these groups permitted estimates to be obtained with the two sexes separately). Individual sub-cohort analyses were meta-analysed.

The estimated contributions of self-reported smoking status (and other covariates) to trait variation in UK Biobank are shown in S2 Table. These were similar to the ones observed in Generation Scotland, varying between 0.2% (for height) and 1.4% (for waist-to-hip ratio).

**Fig 3:**
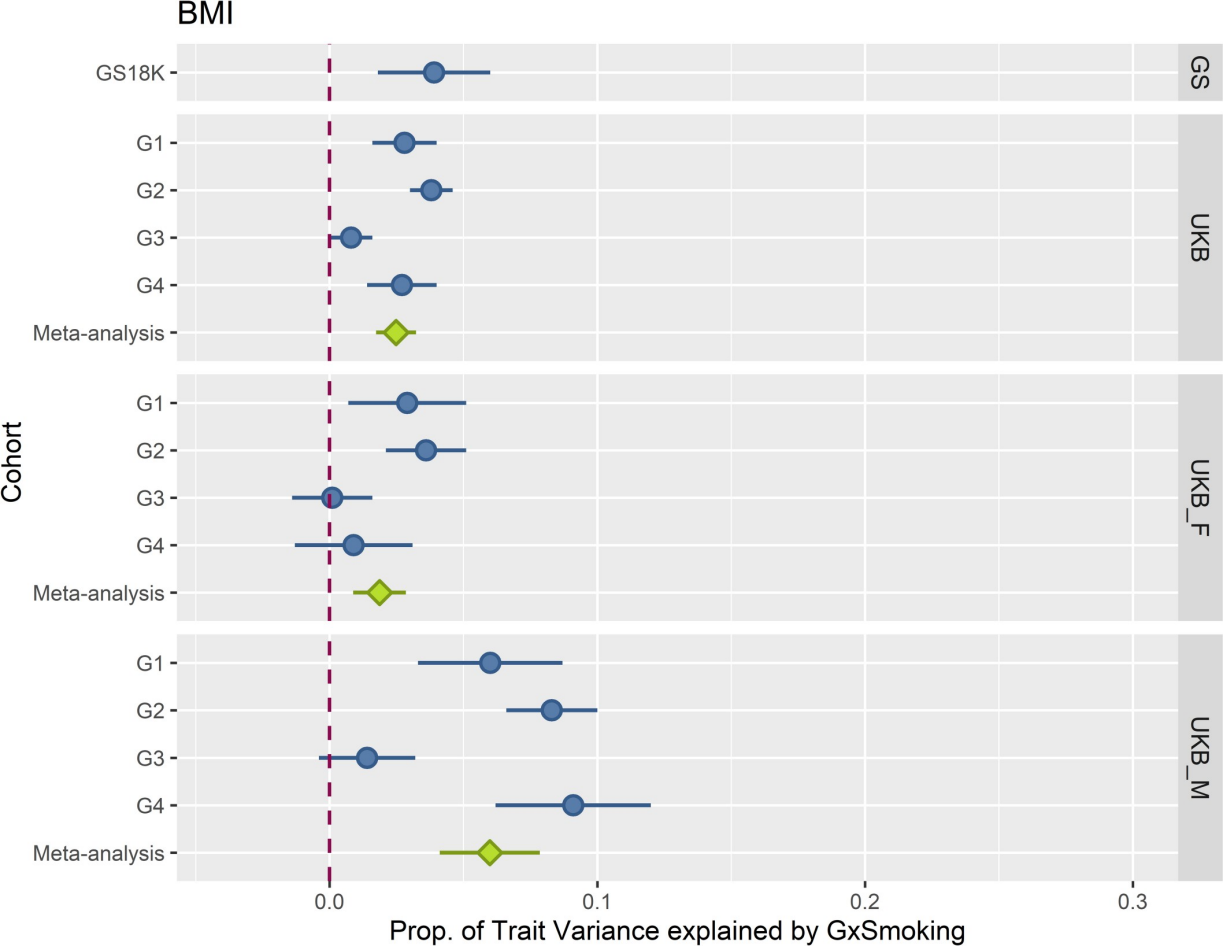
Proportion of BMI variation explained by Genome-by-Smoking interactions across all cohorts and sub-cohorts. The plot shows the proportion of BMI variance (the bars represent standard errors) explained by the genome-by-smoking interaction (x-axis) in the mixed model analyses across cohorts (y-axis). Panels from top to bottom represent cohorts: Generation Scotland (GS), UK Biobank (UKB), UK Biobank females (UKB_F) and UK Biobank males (UKB_M). Blue coloured data points show sub-cohort results, green coloured data points show meta-analyses of the corresponding panel sub-cohorts.

Fig 3 shows the proportion of BMI variance explained by the genome-by-smoking interactions in each of the cohorts and sub-cohorts (Generation Scotland, four UK Biobank groups and the UK Biobank meta-analysis). Results for other traits are displayed in S2 Fig and full details of the analyses for all traits including estimates, standard errors and log-likelihood ratio tests are shown in S4, S5 and S6 Tables. Results for the genome-by-smoking-by-sex interactions are shown in S3 Fig and S7 Table.

Meta-analyses of the sub-cohorts showed significant genome-by-smoking interactions in all traits except for height when analysing both sexes together and males separately, whereas in females, only fat percentage showed a significant effect of the interaction. Similarly, the genome-by-smoking-by-sex interactions were significant for all traits but height. Genome-by-smoking-by-sex interaction effects explained between 2 and 6% of the observed variation.

### Smoking-associated methylation

To explore the value of DNA methylation data as a proxy for environmental variation, we modelled similarity between individuals based on their DNA methylation levels at a subset of 62 CpG sites previously associated with smoking [18,33] and which had heritabilities lower than 40%, aiming to target methylation variation that is predominantly capturing environmental variation (for details see Methods). To show that our models can provide accurate estimates we performed a series of simulations. Details and results for those are shown in S1 Text.

**Fig 4:**
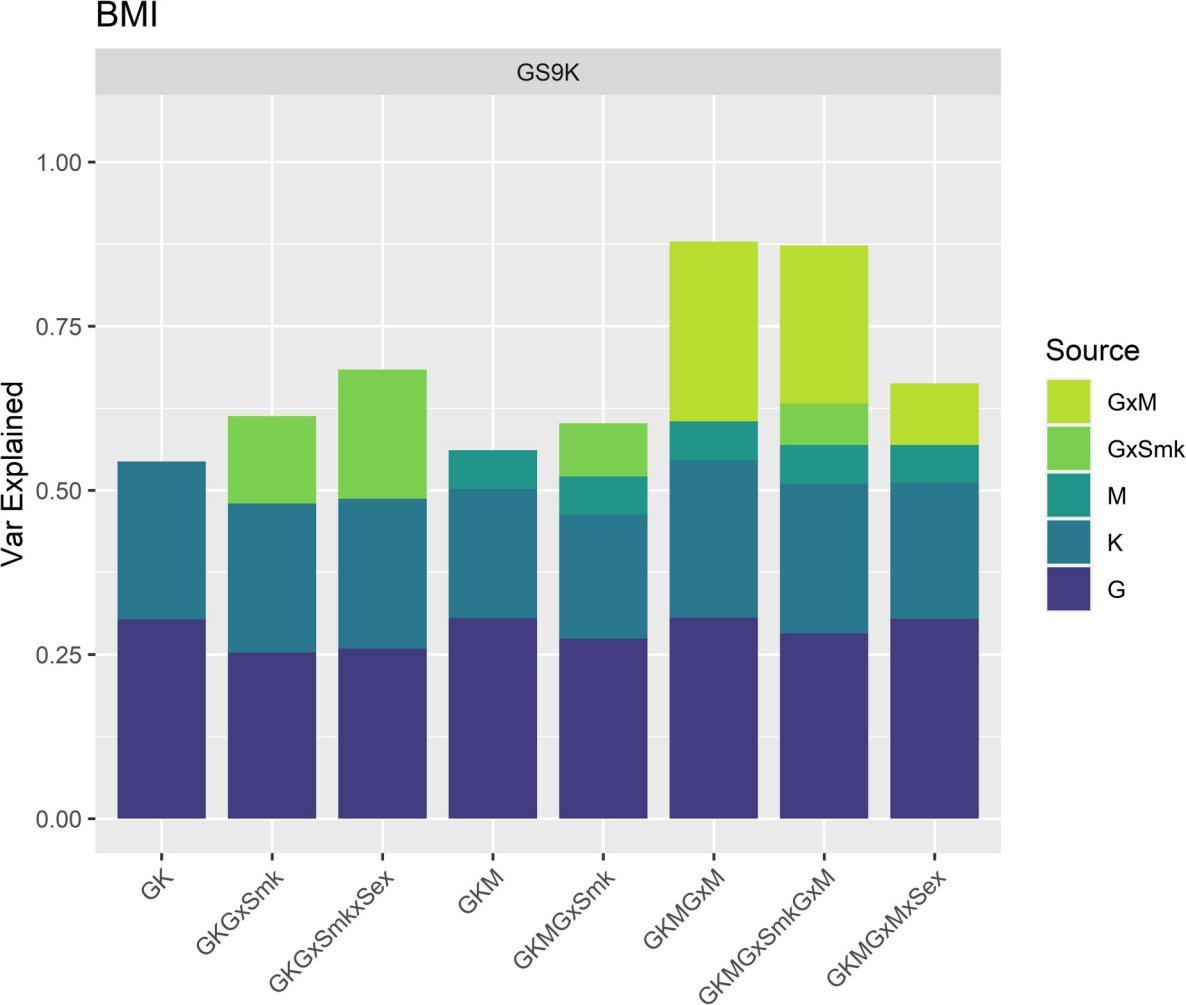
Proportion of BMI variation explained by genetic, environmental and methylation sources in GS9K. Proportion of BMI variance (y-axis) explained by each of the genetic, environmental and interaction sources in the corresponding models (x-axis). G: Genomic, K: Kinship, GxSmk: Genome-by-Smoking, M: Smoking associated methylation, GxM: Genome-by-Methylation, GxSmkxSex: Genome-by-Smoking-by-Sex, GxMxSex: Genome-by-Methylation-by-Sex.

Fig 4 shows the estimates of the proportion of BMI variance explained by different sources included in the mixed linear models in ~9K individuals in Generation Scotland (GS9K - right panel) including models with methylation and genome-by-methylation interactions for models with self-reported smoking status fitted as a fixed effect. Results for other traits are displayed in S1 Fig and full details of the analyses for all traits including estimates, standard errors and log-likelihood ratio tests, and results for smoking status fitted as a random effect are shown in S8 Table. Inclusion of the methylation covariance matrix improved the models for all traits and explained 0.7% of the variance for height and between 3–5% of the variance for obesity-related traits. After including in the model this smoking-associated methylation component, the variation explained by self-reported smoking status dropped to zero for all traits (S8 Table, Model = GKEM), i.e., smoking-associated methylation absorbed the variance explained by the self-reported variable. When exploring the interactions with self-reported smoking status, the estimates in the subset of individuals with methylation data available (N ~ 9K) are substantially larger than in the whole cohort. For example, for BMI, the size of the genome-by-smoking component increased from 4% (GS18K) to 13% (GS9K), however, due to the large standard errors, these two estimates are not significantly different from each other. Inclusion of the genome-by-methylation interaction component nominally improved the model fit for weight, BMI, and waist circumference, with estimates of the interaction component of over 20% of the trait variance. When fitting jointly the two interaction components (genome-by-smoking and genome-by-methylation) the estimates were not significant for either interaction component (or just nominally significant in the case of genome-by-methylation for BMI).

## Discussion

Most complex diseases have moderate heritabilities, with various environmental sources of variation, for example, lifestyle and socioeconomic differences between individuals, also contributing to disease risk [5]. These diseases, particularly obesity, pose major challenges for public health and are associated with heavy economic burdens [3,4,37]. To prevent the problems resulting from complex diseases, effective personalised approaches that help individuals to reach and maintain a healthy lifestyle are required. To achieve that aim, knowledge of environmental effects and gene-by environment interactions (GxE, i.e., understanding the differential effects of an environmental exposure on a trait in individuals with different genotypes [38]) is required. This is a challenge, particularly for environmental factors that are not easy to measure, or that are measured with a lot of error. It has previously been assumed that GxE effects contribute to variation in obesity-related traits [6,8], but the total contribution to trait variation was not known. Previous analyses exploring GxE in obesity, as well as other traits, took advantage of particular individual genetic variants with known effects, or constructed polygenic scores, combining several genetic variants which reflect genetic risks for the individuals [39,40]. Here we analysed contributions of interactions between the genome (as a whole) with smoking, both using self-reported measures of smoking and methylation data as a proxy for smoking.

Our estimates of the effects of genome-by-smoking interactions in obesity-related traits are larger than those estimated in Shin and Lee [16] but in line with Robinson, et al. [12] for BMI. However, our analyses indicate that the magnitude is substantially different in the two sexes, with interactions playing a bigger role in males for most traits studied (weight, waist, hips, fat%). Joint analysis of males and females provides less accurate estimates, suggesting that splitting the sexes or modelling the interactions with sex is a more sensible way of analysing the data. The estimates of the variance explained by the interaction components obtained from the genome-by-methylation analyses were large, with also large standard errors. These results, despite not being significant after multiple correction testing, are potentially interesting and should be investigated further. Some studies have suggested that there is potential confounding between interaction and covariance effects in linear mixed models. The CpG sites used to model the methylation similarity between individuals were previously corrected for genomic effects (see Methods) removing potential covariance between the genetic and methylation effects [41,42].

We estimated that the impact of genome-by-smoking interaction ranges from between 5 to 10% of variation in the studied traits with the exception of height, which we used as a negative control. Our results suggest a larger interaction component in traits associated with weight (BMI, weight, waist, hips) than in those more related to adiposity (waist-to-hip ratio, fat percentage). Biological interpretation of these interactions implies that some genes contributing to obesity differences between individuals have different effects depending on smoking status. This could be mediated in several ways, for example, via genetic variants that affect both obesity and smoking. Some metabolic factors associated with food intake, such as leptin, are suspected to play a role in smoking behaviours, and rewarding effects of food and nicotine are partly mediated by common neurobiological pathways [43]. For example, if these common genetic architectures balance the two behaviours (i.e., more tobacco consumption leading to eating less [43]) the genetic effects of obesity-related traits will be different depending on the smoking status. The interactions could also be driven by gene-by-gene interactions (GxG), i.e., genetic variants affecting obesity modulated by smoking associated genetic variants. Under this scenario smoking status would be capturing smoking associated variants, and the genome-by-smoking interaction would represent GxG instead of GxE. However, given the relatively small heritability of tobacco smoking (SNP heritability ~18% [44]), it is unlikely that all the variation we detected is driven by GxG.

One of the sub-groups of UK Biobank (G3) showed consistently non-significant estimates of the interactions for all traits. The different behaviour for this cohort is not driven by characteristics like the proportion of smokers (S3 Table), or by its genetic stratification. Without any other evidence we cannot attribute these systematic lower estimates to anything but chance.

When we estimated the effect of smoking using the methylomic proxy (62 CpG sites associated with smoking from two independent studies [18,33]), the smoking associated variance increased substantially for all traits (from 2% to 6% for BMI). The methylation component captured the same variance as the self-reported component and some extra variation (S8B Table). This increase in variation captured could be due to a better ability to separate differences between different levels of smoking (e.g., the self-reported status does not include amount of tobacco smoked, while the methylation might be able to capture this information better). These smoking associated CpG sites could also be picking up variation from other environmental sources that are not exclusively driven by smoking, but correlated with it, such as alcohol intake. When checking in the literature for other possible associations between the 62 CpG sites and other environmental measures (S9 Table), 20 of these CpGs have previously been associated with age, 15 with alcohol intake or alcohol dependence, 11 with educational attainment, 10 with different types of cancer; and a few with other diseases [45,46]. Unlike for smoking, for most of these associations with other traits, it is unclear if they are casual, or if they could as well be driven by smoking (e.g., alcohol consumption is associated with smoking and picking up a smoking signal).

The fact that variation in obesity can be explained by CpG sites associated with smoking does not imply a causal effect of smoking or methylation on obesity. Methylation is affected by both genetic and environmental effects. Here we selected a subset of CpG sites with moderate to small heritability (lower than 40%, S9 Table) and we modelled them jointly with a genomic similarity matrix, making it unlikely that the variance picked up by the methylation matrix is genetic in nature. While most changes in methylation at these CpG sites are thought to be causally driven by smoking [18], associations between methylation and other complex traits, such as BMI, are less well characterised and mostly likely to be reversely caused [47] (i.e., BMI affecting methylation), however, since our aim was to use methylation as a proxy for the environment, causality does not impact the conclusion of the study. It is, however, important to notice the variable nature of the methylation data, which will change during the life course of individuals unlike the genetics of the individuals, making the inclusion of methylation, measured far back in time, less relevant in a prediction framework [48]. Although this approach should be useful in other populations, a relevant set of CpG sites should be selected reflecting demographic and ethnic relevant associations [49].

To conclude, we showed that methylation data can be used as a proxy to assess smoking contributions to complex trait variation. We used DNA methylation levels at CpG sites associated with smoking as a proxy for smoking status to assess the contribution of smoking to variation in obesity-related traits. This principle could be extended to take advantage of the wealth of uncovered associations between various *omics* and environmental exposures of interest, particularly for those that are difficult to measure. In humans, relevant interactions could be investigated by exploiting the links between methylation and alcohol intake, metabolomics and diets, the gut microbiome and diets, etc., and expanding to other species, between the gut microbiome and greenhouse emissions in cattle. This could help expanding our knowledge on their contribution to complex phenotypes, and potentially, help understand the underlying biology and to improve prediction and prognosis.

## Methods

### Ethics statement

#### Generation Scotland

Ethical approval for the study was given by the NHS Tayside committee on research ethics (ref: 05/s1401/89). Governance of the study, including public engagement, protocol development and access arrangements, was overseen by an independent advisory board, established by the Scottish government. Research participants gave consent to allow both academic and commercial research.

#### UK Biobank

The study was conducted with the approval of the North-West Research Ethics Committee, in accordance with the principles of the Declaration of Helsinki, and all participants gave written informed consent (https://www.ukbiobank.ac.uk/learn-more-about-uk-biobank/about-us/ethics). Data access to UK Biobank was granted under application 19655.

### Data

#### Generation Scotland

We used data from Generation Scotland: Scottish Family Health Study (GS) [50,51]. Individuals were genotyped with the Illumina HumanOmniExpressExome-8 v1.0 or v1.2. We used PLINK version 1.9b2c [52] to exclude SNPs that had a missingness > 2% and a Hardy-Weinberg Equilibrium test P < 10^−6^. Markers with a minor allele frequency (MAF) smaller than 0.05 were discarded. Duplicate samples, individuals with gender discrepancies and those with more than 2% missing genotypes were also removed. The resulting data set was merged with the 1092 individuals of the 1000 Genomes population [53] and a principal component analysis was performed using GCTA [54]. Individuals more than 6 standard deviations away from the mean of principal component 1 and principal component 2 were removed as potentially having African/Asian ancestry as shown in Amador et al. [55]. After quality control, individuals had genotypes for 519,819 common SNP spread over the 22 autosomes. Of the ~24,000 individuals in GS, the number of individuals with complete information for smoking and other measures included in the models was 18,522 so we used this core set of samples for the analyses in order to allow comparisons between the models, we refer to this set of samples as GS18K.

#### UK Biobank

The UK Biobank database include 502,664 participants, aged 40–69, recruited from the general UK population across 22 centres between 2006 and 2010 [56]. They underwent extensive phenotyping by questionnaire and clinic measures and provided a blood sample. Phenotypes and genotypes were downloaded direct from UK Biobank. UK Biobank participants were genotyped on two slightly different arrays and quality control was performed by UK Biobank. The two are Affymetrix arrays with 96% of SNPs overlap between both. Further information about the quality control can be found in the UK Biobank website (https://www.ukbiobank.ac.uk/register-apply/). Only genetically white British individuals were used in the analyses. The total number of individuals with complete information for measures of interest was 374,453. Genotypes were available for 534,427 common markers spread over the 22 autosomes.

For computational reasons, UKB individuals were split in four sub-cohorts to be analysed separately. The grouping was based in latitudinal differences between the assessment centres the individuals attended. Number of individuals and assessment centres are shown in S3 Table.

### Phenotypes

#### Generation Scotland

We used measured phenotypes for eight traits: height, weight, body mass index (BMI, computed as weight/height^2^), waist circumference (waist), hip circumference (hips), waist-to-hip ratio (WHR, computed as waist/hips), bio-impedance analysis fat (fat%), and HDL cholesterol. Phenotypes with values greater or smaller than the mean ± 4 standard deviations (after transformation and adjusting for sex, age and age^2^) were set to missing. The traits were pre-adjusted for the effects of sex, age, age^2^, clinic where the measures were taken, and a rank-based inverse normal transformation was performed on the residuals. These values were used in all the analyses.

#### UK Biobank

We used measured phenotypes for anthropometric traits: height, weight, body mass index (BMI, computed as weight/height^2^), waist circumference (waist), hip circumference (hips), waist-to-hip ratio (WHR, computed as waist/hips), body fat percentage (fat%)Phenotypes with values greater or smaller than the mean ± 4 standard deviations (after transformation and adjusting for sex, age and age^2^) were set to missing. The traits were pre-adjusted for the effects of sex, age, age^2^, clinic where the measures were taken, and a rank-based inverse normal transformation was performed on the residuals. These values were used in all the analyses.

### Smoking status

We used self-reported smoking status on both cohorts. Individuals were classified with respect of smoking as “never smoked”, “ex-smoker” and “current smoker” for Generation Scotland, and as “never smoked”, “ex-smoker”, “current smoker”, and “occasional smoker” for UK Biobank. The number of individuals in each category are shown in S3 Table.

### DNA methylation data

DNA methylation data is available for a subset of 9,537 participants from the GS cohort, as part of the Stratifying Resilience and Depression Longitudinally (STRADL) project [57]. From those, we used N = 8,821 individuals that had complete information for all the same set of measures as used in the smoking status analysis. We refer to this subset of individuals as GS9K. DNA methylation was measured at 866,836 CpGs from whole blood genomic DNA, using the Illumina Infinium MethylationEPIC array. Quality control was performed using R (version 3.6.0) [58], and packages *shinyMethyl* [59] and *meffil* [60]. We removed outliers based on overall array signal intensity and control probe performance and samples showing a mismatch between recorded and predicted sex. We removed samples with more than 0.5% of sites with a detection p-value of > 0.01; and probes with more than 5% samples with a bead count smaller than 3. Normalization was performed using the R package *minfi* [61], that produced methylation M-values that were used in downstream analyses. For each methylation site, two linear mixed model were used to remove effects of technical and biological factors correcting for technical variation, i.e., Sentrix id, Sentrix position, batch, clinic, appointment date, year and weekday of the blood extraction, and 20 principal components of the control probes; and biological variation, i.e., sex, age, estimated cell proportions (CD8T, CD4T, NK, B Cell, Mono, and Gran cells proportions based on Houseman, et al. [62]), and two genetic (Genetic and Kinship) and three common environment (Family, Couples, Siblings) effects. For more information see Xia et al [36] and Zeng et al [17]. The residual values of those corrections were used for subsequent analyses.

#### Smoking associated CpG sites

We selected a subset of CpG sites identified in two epigenome-wide association studies of tobacco consumption [18,33]. We selected CpG sites with a p-value lower than 10^−7^ in both Ambatipudi et al. [18] (associations between CpG sites and differences between groups: smokers *v* non-smokers, smokers *v* ex-smokers, ex-smokers *v* non-smokers) and in Joehanes et al. [33] (associations between CpG sites and dosage of tobacco smoked) to obtain a subset of CpG sites confidently associated with smoking (i.e., from two sources). We identified those CpG sites with heritabilities lower than 40% in Generation Scotland (as measured in the last step of the quality control of the data, see below) that are available in Generation Scotland. The list of 62 CpG sites is available in S9 Table.

### Covariance matrices

To model the different sources of variance we used a set of covariance matrices representing similarity between individuals based on genetic components, environmental components, or both.

#### Genetic matrices

**G** is a genomic relationship matrix (GRM) reflecting the genetic similarity between individuals [63,64]. **K** is a matrix representing pedigree relationships as in Zaitlen et al. [65]. It is a modification of **G** obtained by setting those entries in **G** lower than 0.025 to 0.

#### Smoking matrices

**SMK** is a matrix representing common environmental effects shared between individuals with same smoking status i.e., **SMK** contains a value of 1 between individuals in the same smoking category and a 0 between individuals in different categories.

#### Gene-environment interaction matrices

**GxSmk** is a matrix representing genome-by-smoking interactions. It was computed as the cell-by-cell product (Hadamard or Schur product) of the corresponding **G** and **SMK** matrices. For an element of the **GxSmk** matrix, if the corresponding **G** or the **SMK** elements are close to zero, the **GxSmk** term will be zero or close to zero as well. Therefore, similarity between individuals due to the interactions represented in the **GxSmk** matrices requires similarity at both genetic and environmental level. This method resembles a reaction norm modelling approach [66].

#### Methylation-derived matrices

**M** is a matrix representing similarity between individuals based on DNA methylation levels at 62 smoking associated CpG sites (see *Smoking associated CpG sites* above). A similarity matrix was created using OSCA v 0.45 [67] using algorithm 3 (i.e., iteratively standardizing probes and individuals). **GxM** is a genome-by-smoking interaction matrix computed as a Hadamard product of **G** and **M**.

### Analyses

We performed several variance component analyses using GCTA [54], based in the following linear mixed models:

$$y=X\beta +g_{g}+g_{kin}+\varepsilon$$ (1)

$$y=X\beta +g_{g}+g_{kin}+w+\varepsilon$$ (2)

$$y=X\beta +g_{g}+g_{kin}+w+gw+\varepsilon$$ (3)

$$y=X\beta +g_{g}+g_{kin}+gw+\varepsilon$$ (4)

where *y* is an *n* × 1 vector of observed phenotypes with *n* being the number of individuals, β is a vector of fixed effects and **X** is its design matrix, *g*_*g*_ is an *n* × 1 vector of the total additive genetic effects of the individuals captured by genotyped SNPs with *g*_*g*_ ~ *N*(0, **G***σ*^*2*^_*g*_); *g*_*kin*_ is an *n* × 1 vector of the extra genetic effects associated with the pedigree for relatives with *g*_*kin*_ ~ *N*(0, **K***σ*^*2*^_*k*_). *w* is a *n* × 1 vector representing the common environmental effects of smoking, with *w* ~ *N*(0, **SMK***σ*^*2*^_*w*_). *gw* is a *n* × 1 vector representing interactions between markers and environments with *gw* ~ *N*(0, **GxSmk***σ*^*2*^_*gw*_). *ε* is an *n* × 1 vector for the residuals. The four basic models shown above were expanded to include all combinations of random and fixed effects shown in Fig 1.

The estimates for variance explained by the genome-by-smoking components in the four sub-cohorts of UK Biobank were meta-analysed using the R [58] package *metafor* [68].

## Supporting information

S1 Fig Proportion of trait variation explained by the different sources in Generation Scotland (GS) in each of the eight traits studied. Proportion of trait variance (y-axis) explained by each of the genetic, environmental and interaction sources in the corresponding models (x-axis). Left panel: GS data (Nind~18K) with complete environmental information. Right panel: GS data with methylation information (Nind~9K). G: Genomic, K: Kinship, GxSmk: Genome-by-Smoking, M: Smoking associated methylation, GxM: Genome-by-Methylation, GxSmkxSex: Genome-by-Smoking-by-Sex, GxMxSex: Genome-by-Methylation-by-Sex.
(PDF)

S2 Fig Proportion of trait variation explained by Genome-by-Smoking interactions across all cohorts and sub-cohorts in each of the eight traits studied. The plot shows the proportion of trait variance (the bars represent standard errors) explained by the genome-by-smoking interaction (x-axis) in the mixed model analyses across cohorts (y-axis). Panels from top to bottom represent cohorts: Generation Scotland (GS), UK Biobank (UKB), UK Biobank females (UKB_F) and UK Biobank males (UKB_M). Blue coloured data points show sub-cohort results (GS18K and UKB subgroups G1-G4), green coloured data points show meta-analyses of the corresponding panel sub-cohorts.
(PDF)

S3 Fig Proportion of trait variation explained by Genome-by-Smoking-by-Sex interactions across all cohorts and sub-cohorts in each of the eight traits studied. The plot shows the proportion of BMI variance (the bars represent standard errors) explained by the genome-by-smoking-by-sex interaction (x-axis) in the mixed model analyses across cohorts (y-axis). Panels from top to bottom represent cohorts: Generation Scotland (GS), UK Biobank (UKB), UK Biobank females (UKB_F) and UK Biobank males (UKB_M). Blue coloured data points show sub-cohort results (GS18K and UKB subgroups G1-G4), green coloured data points show meta-analyses of the corresponding panel sub-cohorts.
(PDF)

S1 Table Results for all models for GS18K cohort. A. Models with smoking fitted as a random effect. B. Models with smoking fitted as a random effect. The tables show, for each trait, proportion of the phenotypic variance explained (Var), standard error (SE), Significance of the t-statistic (Sig, P), P value for the log-likelihood ratio test (LRT P, only for the interactions) by each of the components in the model: Genetic (G), Kinship (K), Smoking (when fitted as a random effect, Smk), Genome-by-Smoking interaction (GxSmk), Genome-by-Smoking-by-Sex interaction (GxSmkxSex), Kinship-by-Smoking interaction (KxSmk). Highlighted P values indicate nominally significant results for the interaction components.
(XLSX)

S2 Table Variance explained by fixed effects. Percentage of the phenotypic variance explained by the fixed effects included in the models for each trait and cohort.
(XLSX)

S3 Table Cohorts summaries. Summary statistics (number of individuals in each category or mean values) for the fixed effects included in the models for each of the analysed cohorts.
(XLSX)

S4 Table Results for all models for the four UKB cohorts (joint sexes). The tables show, for each trait, proportion of the phenotypic variance explained (Var), standard error (SE), Significance of the t-statistic (Sig, P), P value for the log-likelihood ratio test (LRT P, only for the interactions) by each of the components in the model: Genetic (G), Kinship (K), Genome-by-Smoking interaction (GxSmk). Highlighted P values indicate nominally significant results for the interaction components in each of the four sub-cohorts of UK Biobank (G1, G2, G3, G4) and their Meta-Analyses.
(XLSX)

S5 Table Results for all models for the four UKB cohorts (males). The tables show, for each trait, proportion of the phenotypic variance explained (Var), standard error (SE), Significance of the t-statistic (Sig, P), P value for the log-likelihood ratio test (LRT P, only for the interactions) by each of the components in the model: Genetic (G), Kinship (K), Genome-by-Smoking interaction (GxSmk). Highlighted P values indicate nominally significant results for the interaction components in males from each of the four sub-cohorts of UK Biobank (G1_M, G2_M, G3_M, G4_M) and their Meta-Analyses.
(XLSX)

S6 Table Results for all models for the four UKB cohorts (females). The tables show, for each trait, proportion of the phenotypic variance explained (Var), standard error (SE), Significance of the t-statistic (Sig, P), P value for the log-likelihood ratio test (LRT P, only for the interactions) by each of the components in the model: Genetic (G), Kinship (K), Genome-by-Smoking interaction (GxSmk). Highlighted P values indicate nominally significant results for the interaction components in females from each of the four sub-cohorts of UK Biobank (G1_F, G2_F, G3_F, G4_F) and their Meta-Analyses.
(XLSX)

S7 Table Results for all models for the four UKB cohorts (joint GxSmkxSex interactions). The tables show, for each trait, proportion of the phenotypic variance explained (Var), standard error (SE), Significance of the t-statistic (Sig, P), P value for the log-likelihood ratio test (LRT P, only for the interactions) by each of the components in the model: Genetic (G), Kinship (K), Genome-by-Smoking-by-Sex interaction (GxSmkxSex). Highlighted P values indicate nominally significant results for the interaction components in each of the four sub-cohorts of UK Biobank (G1, G2, G3, G4) and their Meta-Analyses.
(XLSX)

S8 Table Results for all models for GS9K cohort. A. Models with smoking fitted as a random effect. B. Models with smoking fitted as a random effect. The tables show, for each trait, proportion of the phenotypic variance explained (Var), standard error (SE), Significance of the t-statistic (Sig, P), P value for the log-likelihood ratio test (LRT P, only for the interactions) by each of the components in the model: Genetic (G), Kinship (K), Smoking (when fitted as a random effect, Smk), Genome-by-Smoking interaction (GxSmk), Genome-by-Smoking-by-Sex interaction (GxSmkxSex), Kinship-by-Smoking interaction (KxSmk). Highlighted P values indicate nominally significant results for the interaction components.
(XLSX)

S9 Table Smoking associated CpG sites information. Name, chromosome, location, heritability, and trait associations of the 62 CpG sites associated with smoking. Trait associations were extracted from the EWAS Atlas database.
(XLSX)

S1 Text Model accuracy simulations. Description of the phenotypic simulation process, scenarios, models tested, and simulation results to assess the accuracy of the models used in the real data.
(PDF)
