# Supplementary figures and images for: Genome-wide methylation data improves dissection of the effect of smoking on body mass index
Source: PLoS Genet. 2021 Sep 9;17(9):e1009750. doi: 10.1371/journal.pgen.1009750 (PMC8428545; doi:10.1371/journal.pgen.1009750)

## Height

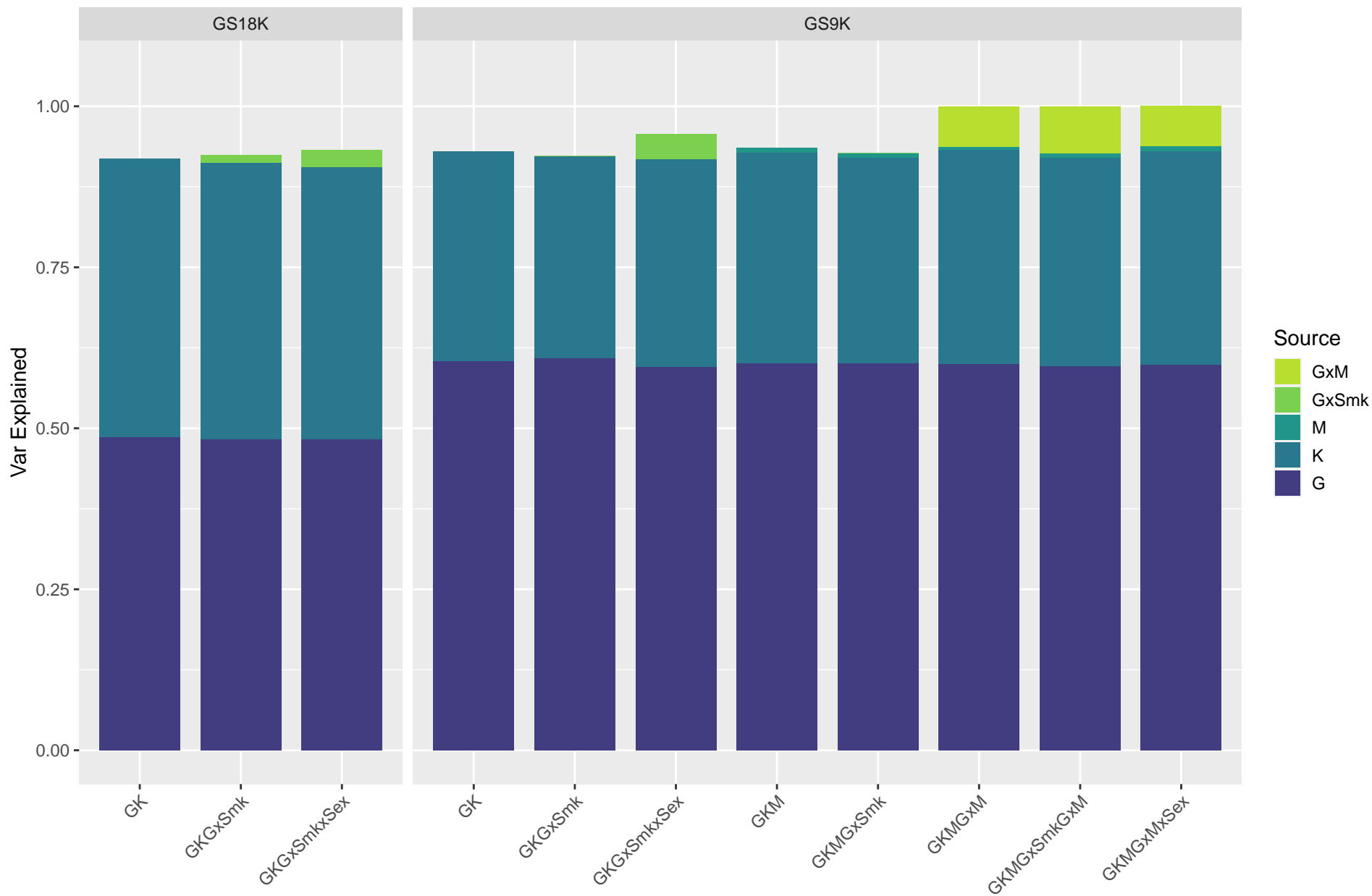

Weight

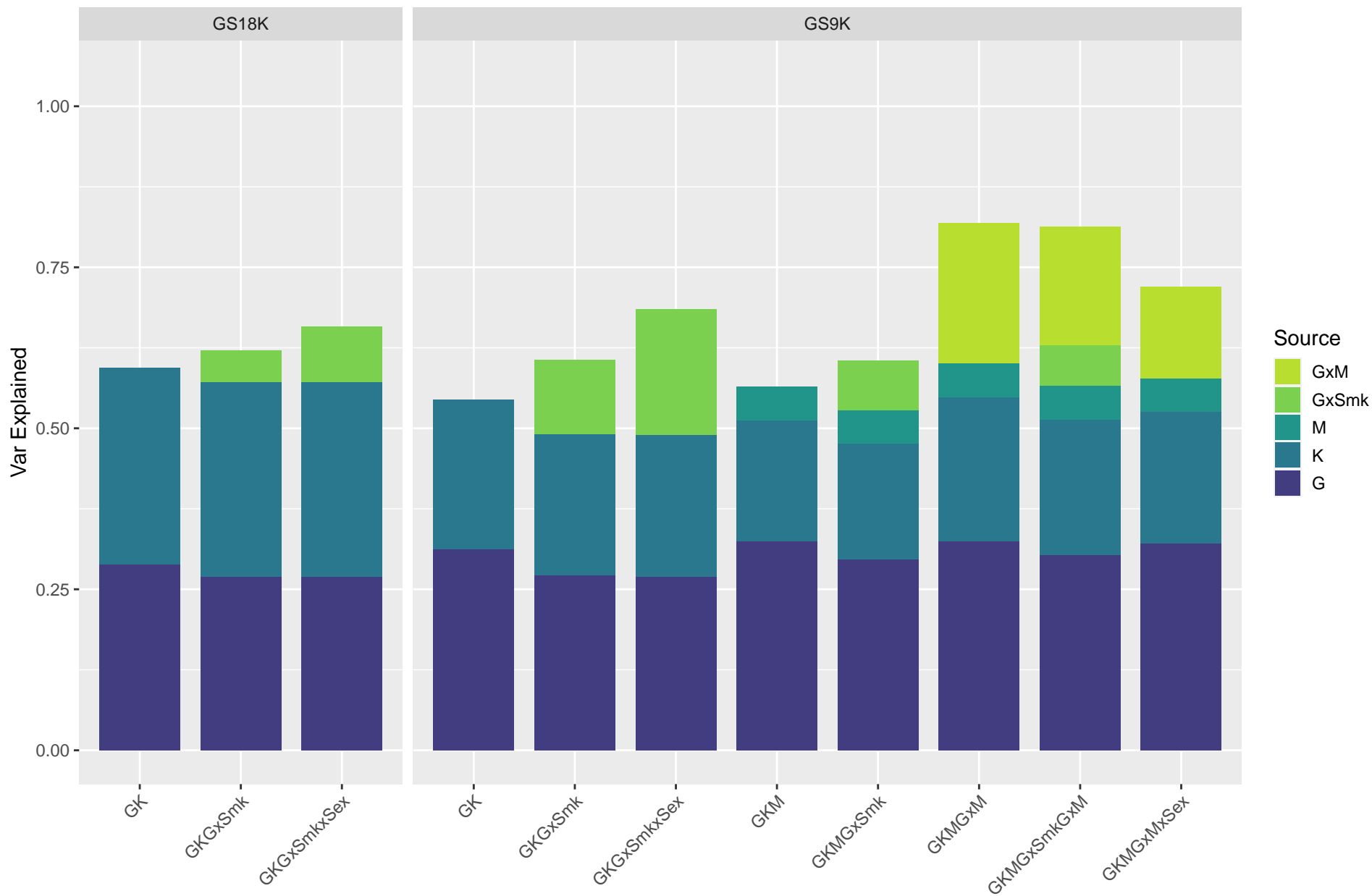

BMI

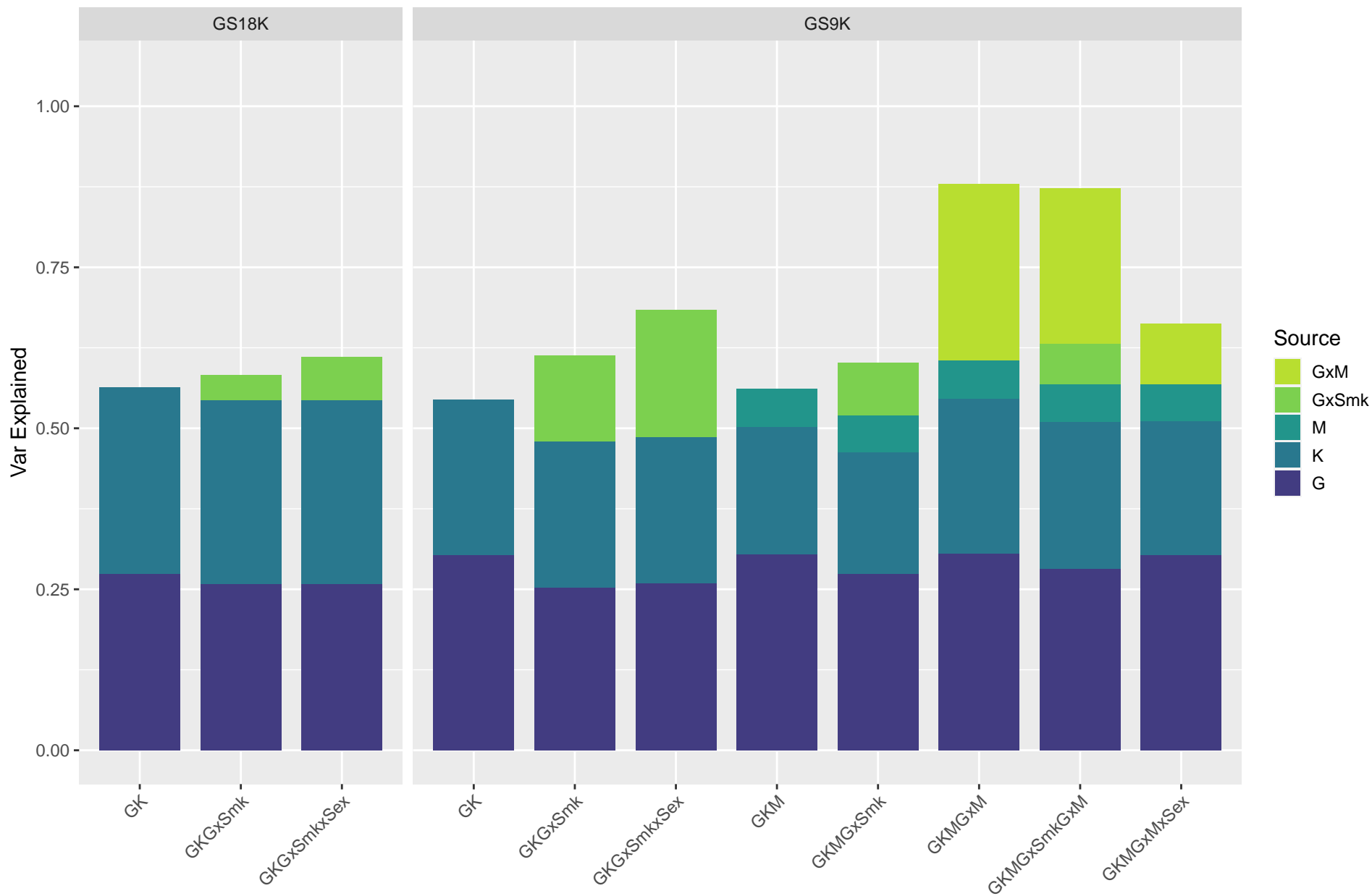

Waist

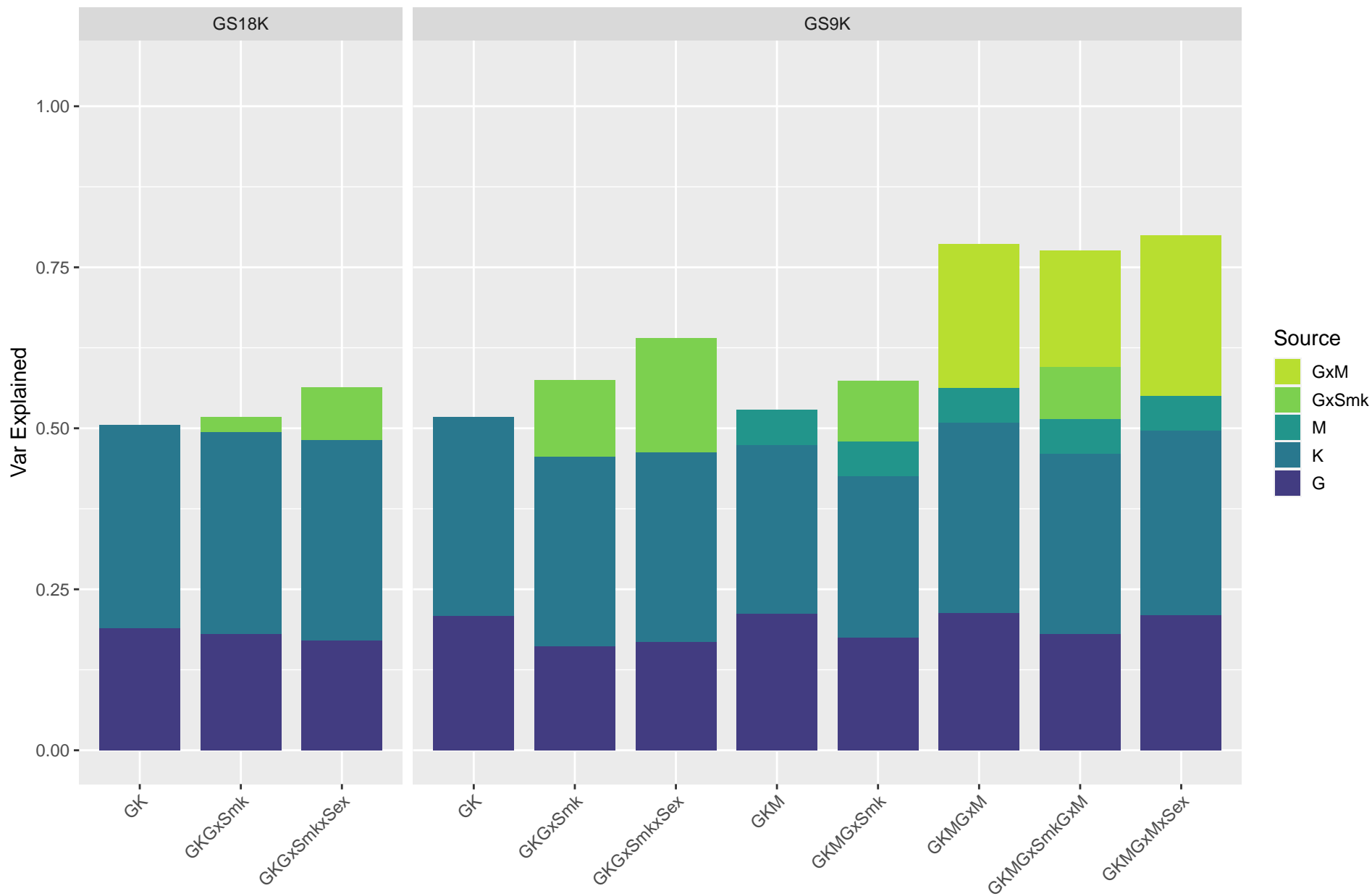

# Hips

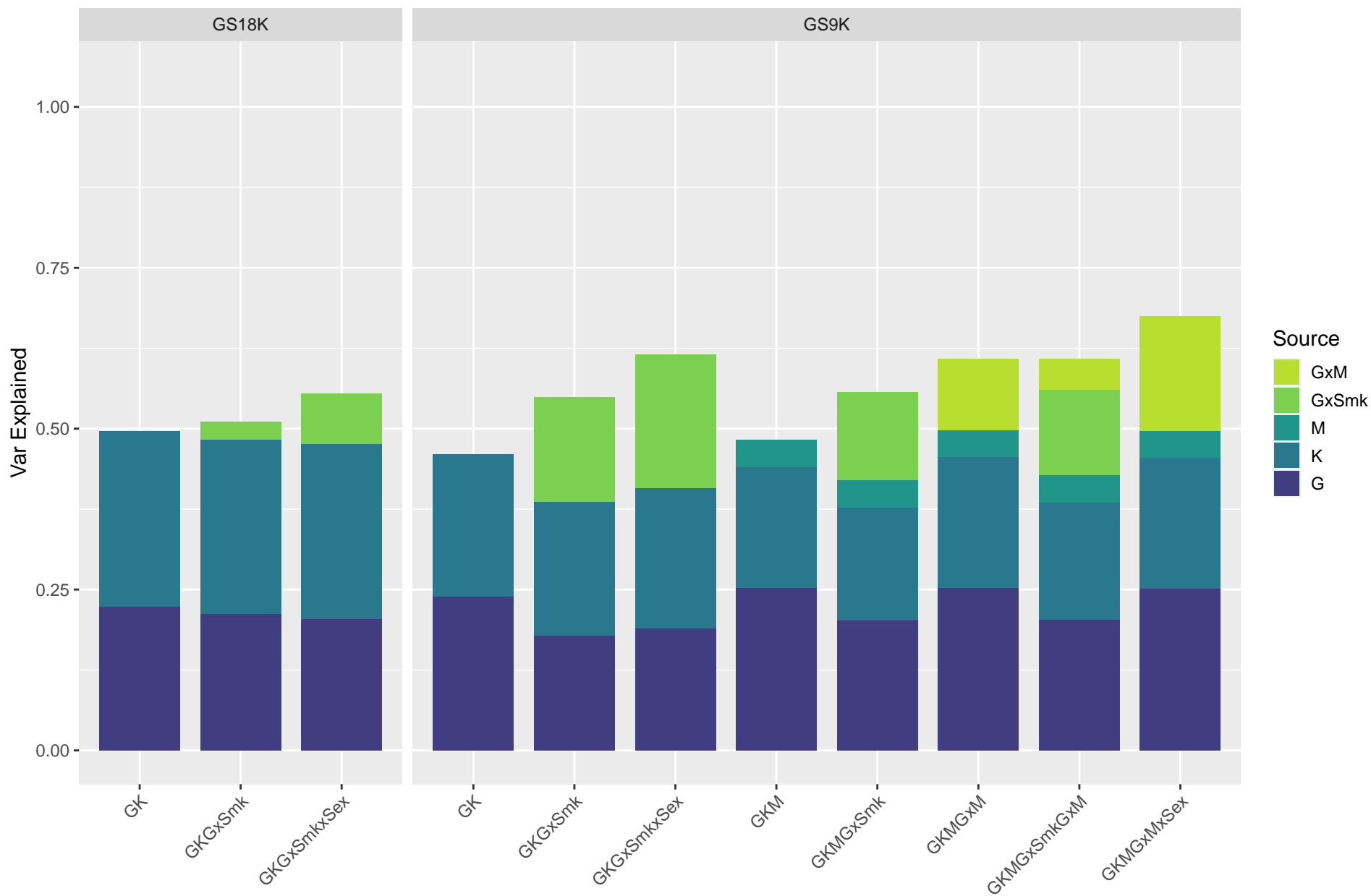

WHR

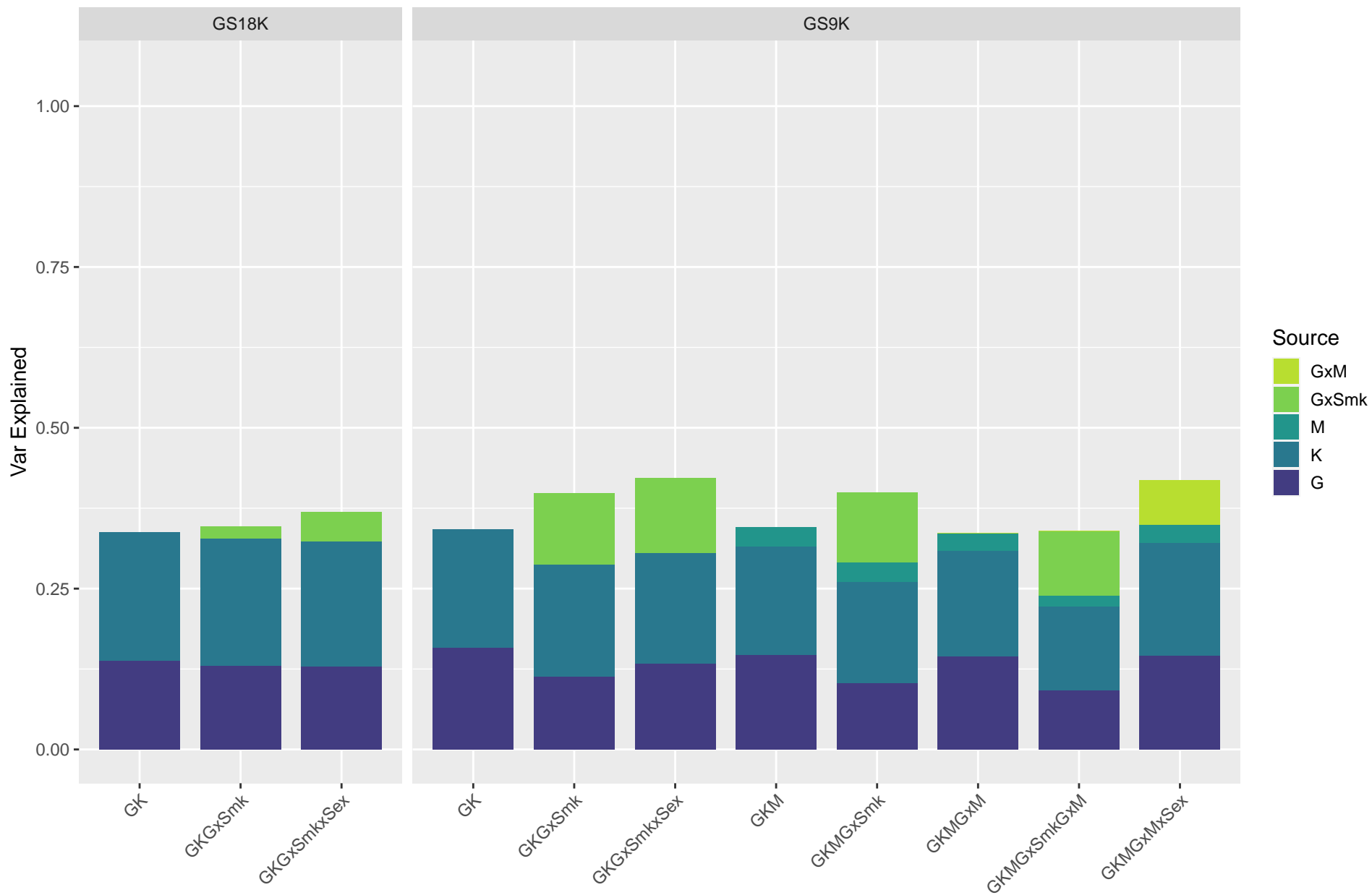

# Fat Percentage

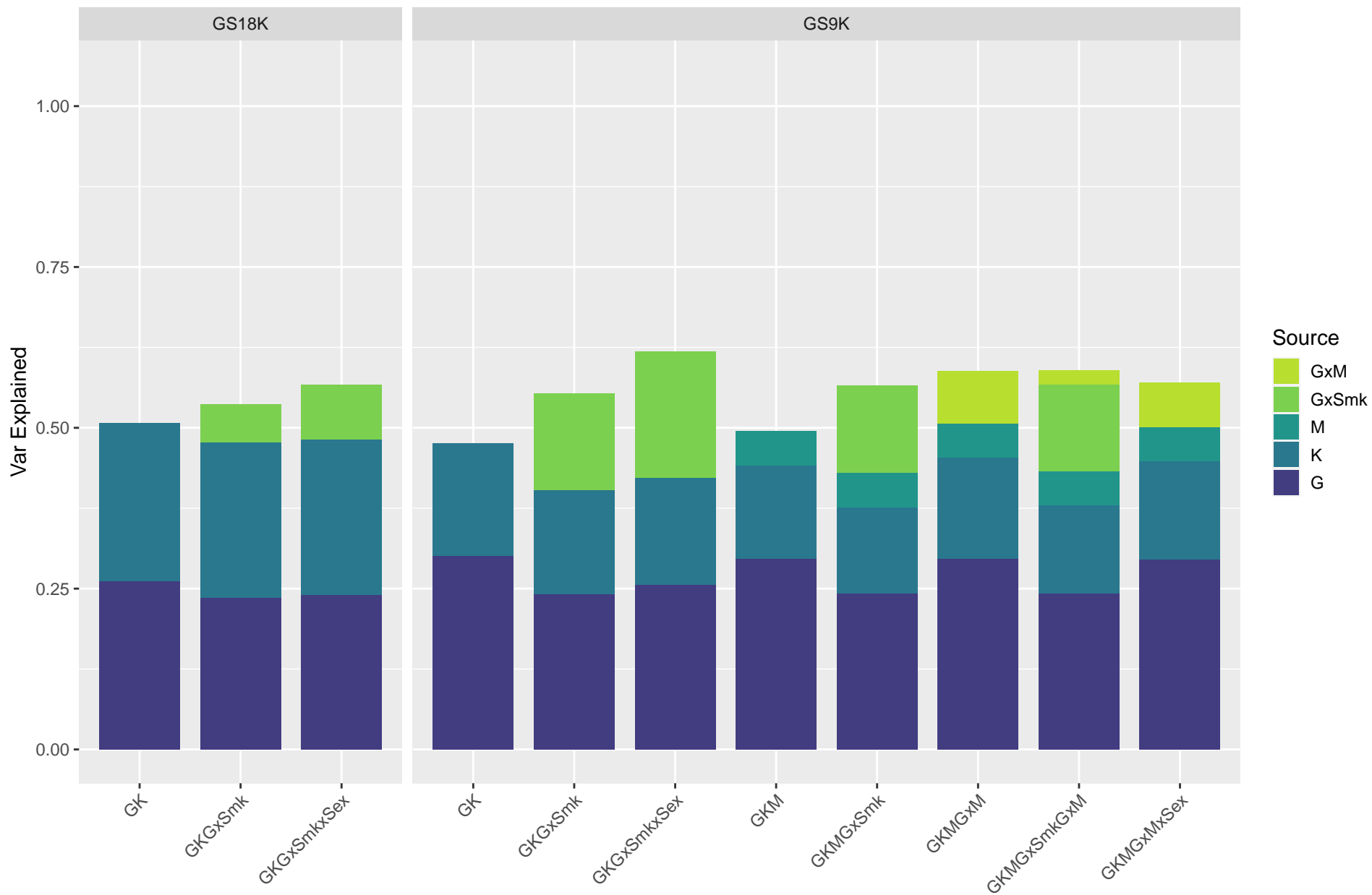

HDL

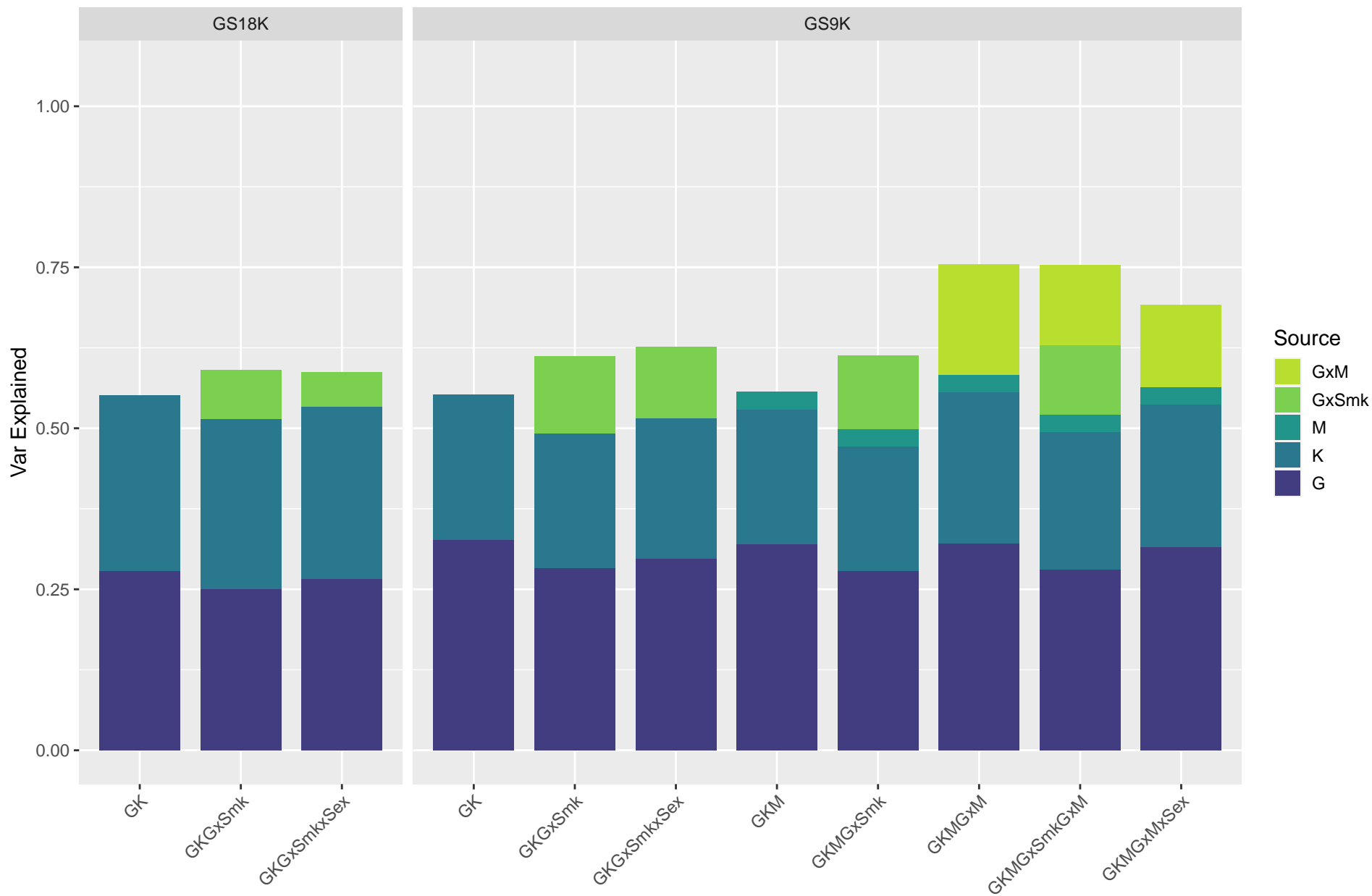

Supplement: S1 Fig — Proportion of trait variance (y-axis) explained by each of the genetic, environmental and interaction sources in the corresponding models (x-axis). Left panel: GS data (Nind~18K) with complete environmental information. Right panel: GS data with methylation information (Nind~9K). G: Genomic, K: Kinship, GxSmk: Genome-by-Smoking, M: Smoking associated methylation, GxM: Genome-by-Methylation, GxSmkxSex: Genome-by-Smoking-by-Sex, GxMxSex: Genome-by-Methylation-by-Sex. (PDF) [file pgen.1009750.s001.pdf]
