# Supplementary material for: Genome-wide methylation data improves dissection of the effect of smoking on body mass index
Source: PLoS Genet. 2021 Sep 9;17(9):e1009750. doi: 10.1371/journal.pgen.1009750 (PMC8428545; doi:10.1371/journal.pgen.1009750)
Supplement: S2 Fig — The plot shows the proportion of trait variance (the bars represent standard errors) explained by the genome-by-smoking interaction (x-axis) in the mixed model analyses across cohorts (y-axis). Panels from top to bottom represent cohorts: Generation Scotland (GS), UK Biobank (UKB), UK Biobank females (UKB_F) and UK Biobank males (UKB_M). Blue coloured data points show sub-cohort results (GS18K and UKB subgroups G1-G4), green coloured data points show meta-analyses of the corresponding panel sub-cohorts. (PDF) [file pgen.1009750.s002.pdf]

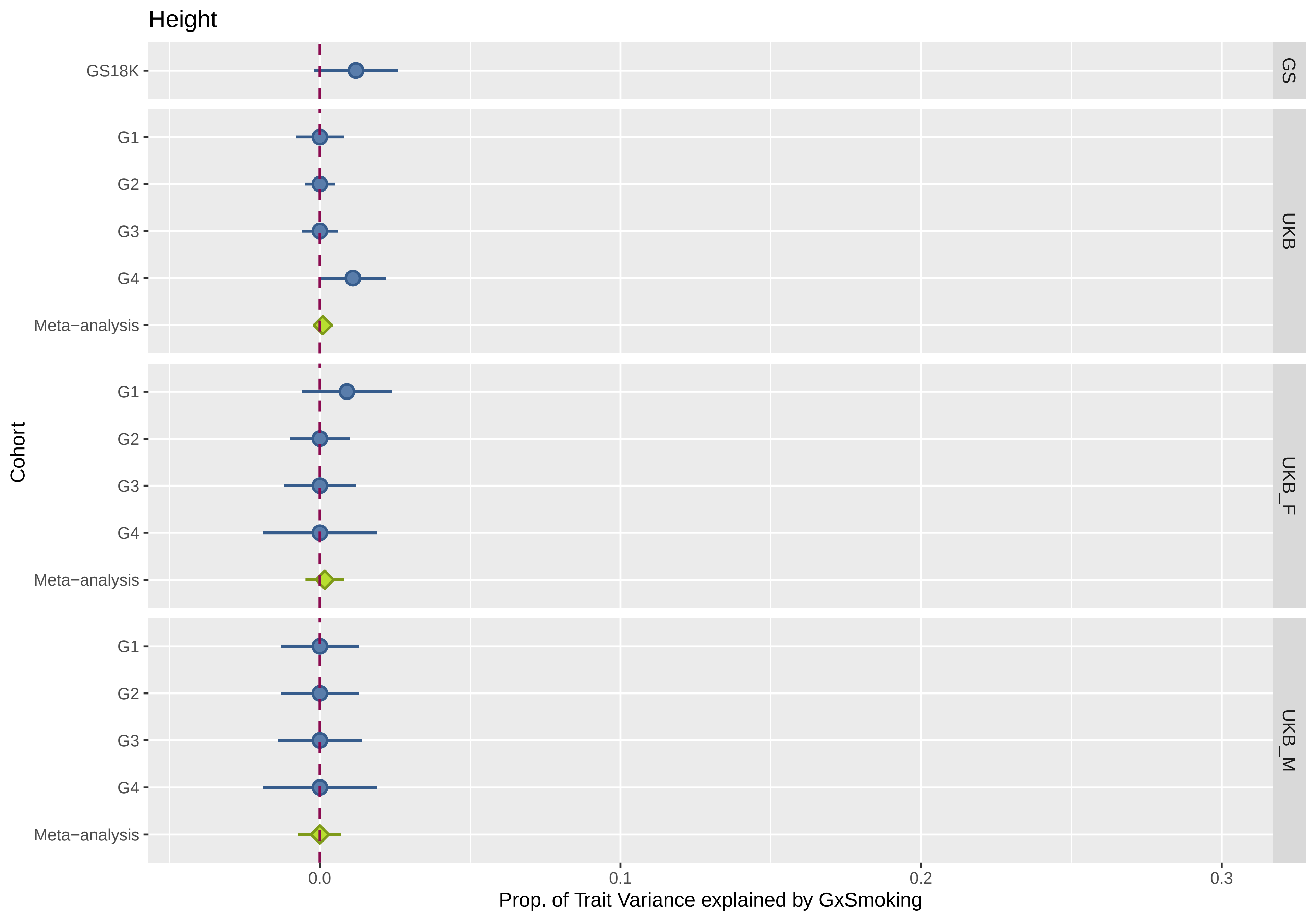
Height
GS18K
G1
G2
G3
G4
Meta−analysis
G1
G2
G3
G4
Meta−analysis
G1
G2
G3
G4
Meta−analysis
GS
UKB
UKB_F
UKB_M
Cohort
0.0
0.1
0.2
0.3
Prop. of Trait Variance explained by GxSmoking

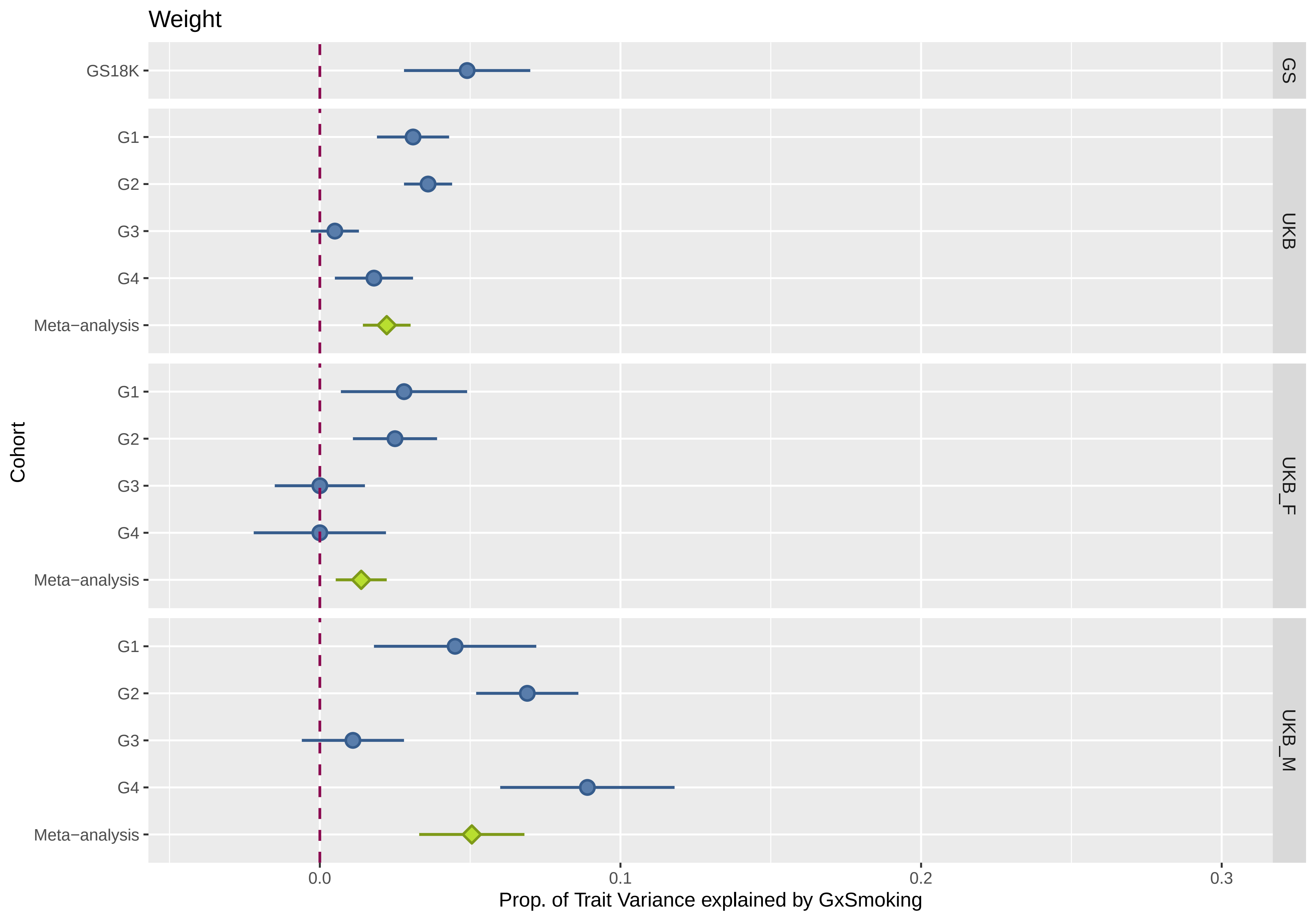

Weight
GS18K
G1
G2
G3
G4
Meta−analysis
G1
G2
G3
G4
Meta−analysis
G1
G2
G3
G4
Meta−analysis
GS
UKB
UKB_F
UKB_M
Cohort
0.0
0.1
0.2
0.3
Prop. of Trait Variance explained by GxSmoking

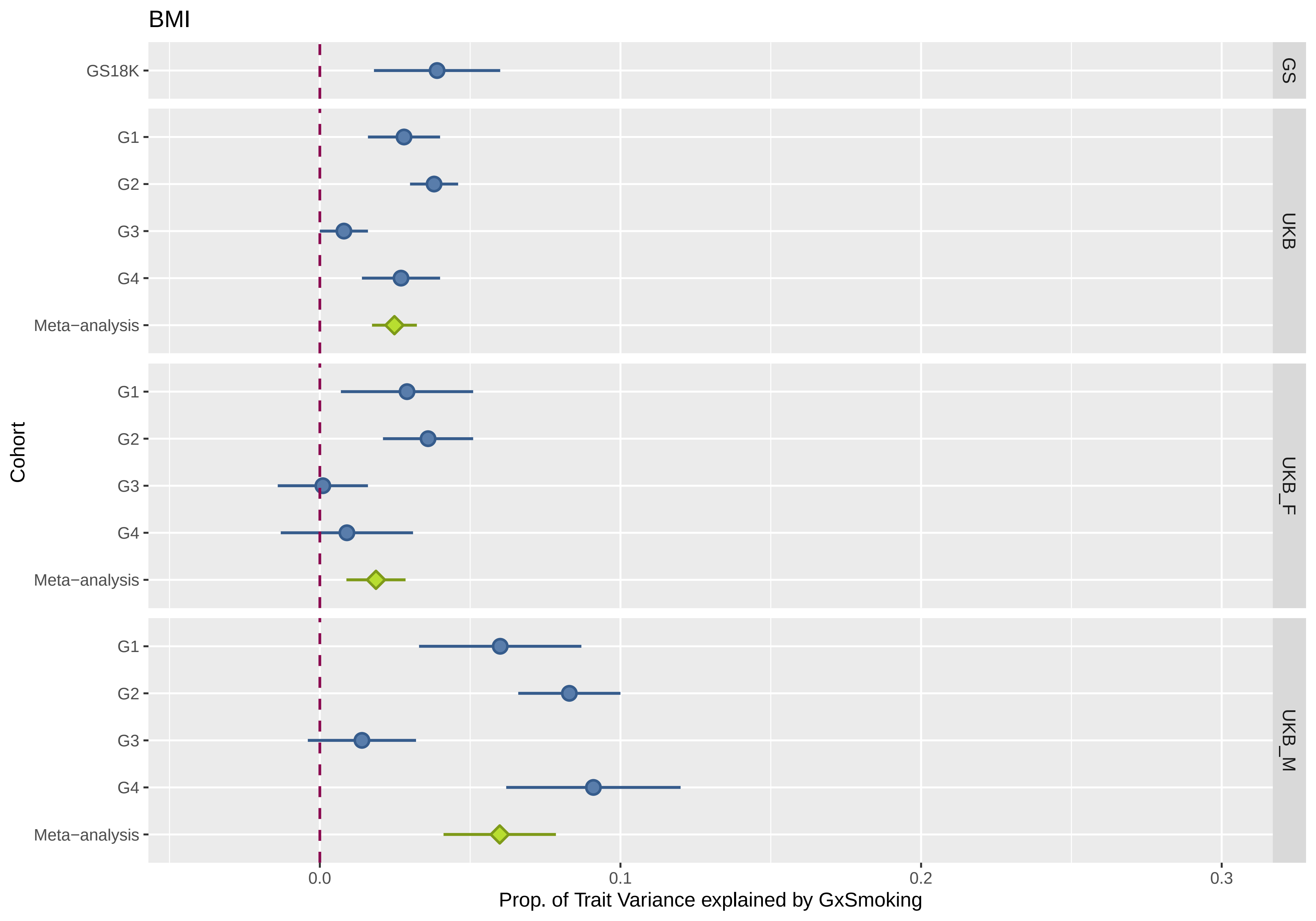

BMI
GS18K
G1
G2
G3
G4
Meta−analysis
GS
UKB
UKB_F
UKB_M
Cohort
0.0
0.1
0.2
0.3
Prop. of Trait Variance explained by GxSmoking

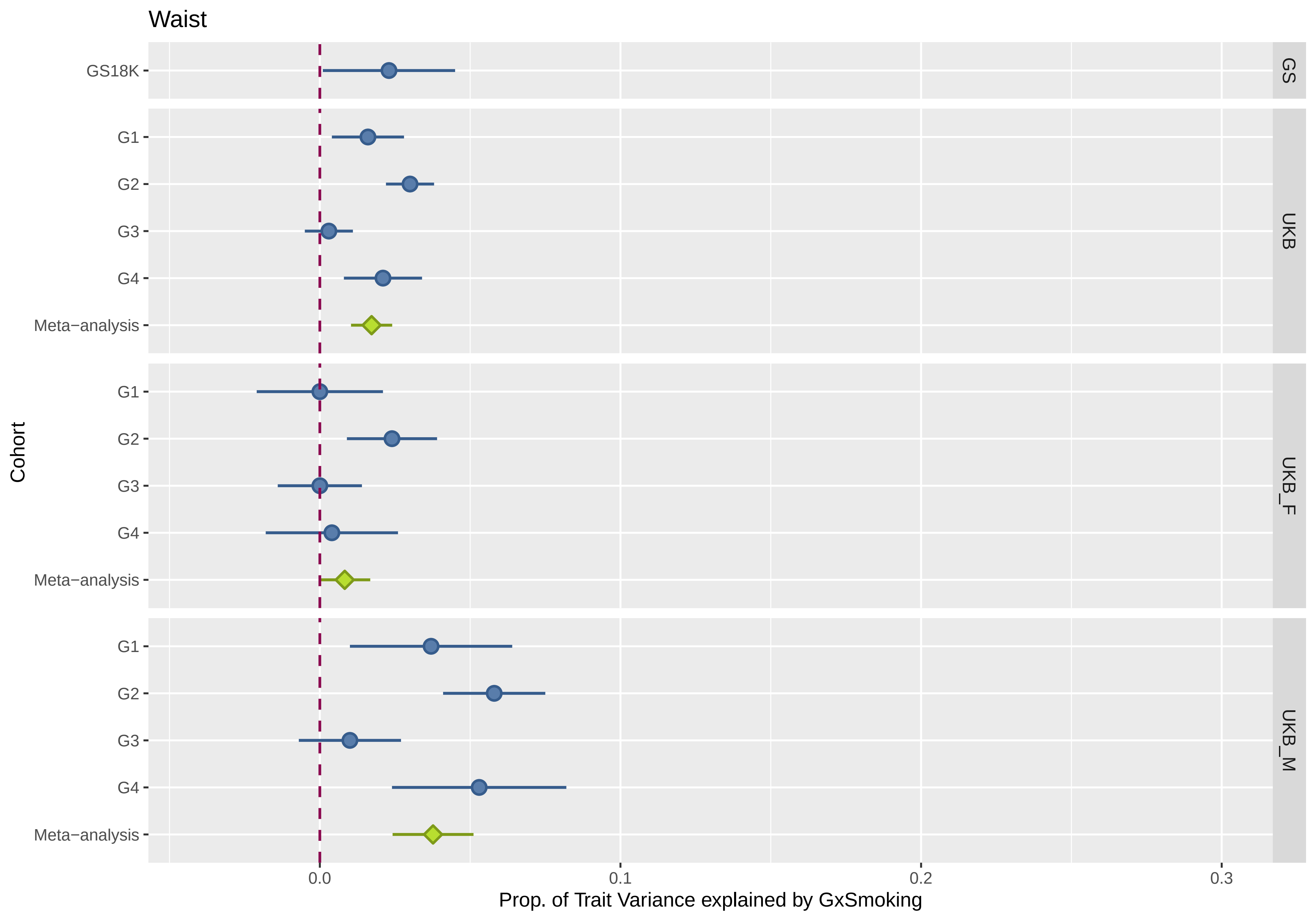
Waist
GS18K
G1
G2
G3
G4
Meta−analysis
G1
G2
G3
G4
Meta−analysis
G1
G2
G3
G4
Meta−analysis
GS
UKB
UKB_F
UKB_M
Cohort
0.0
0.1
0.2
0.3
Prop. of Trait Variance explained by GxSmoking

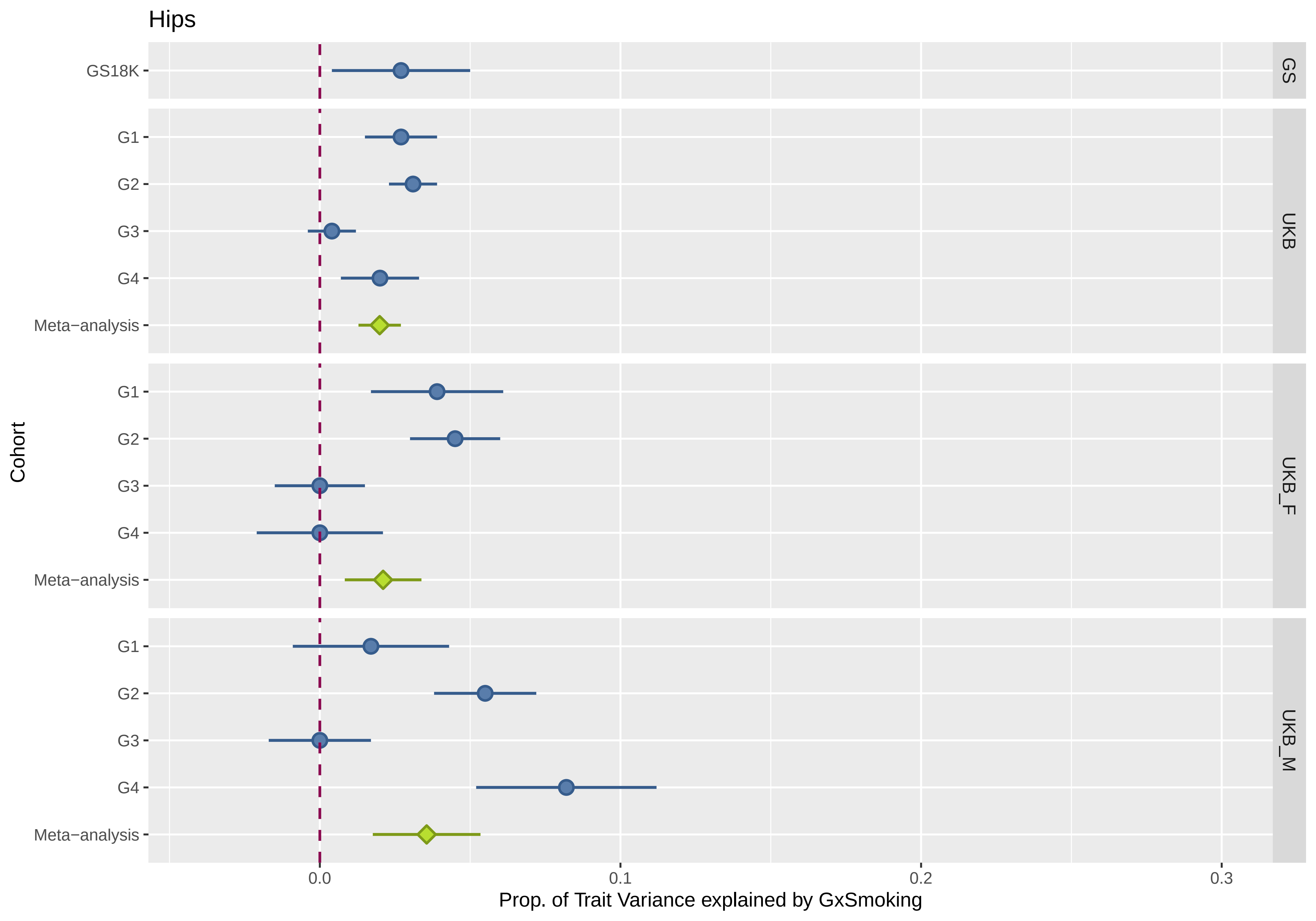

Hips
GS18K
G1
G2
G3
G4
Meta−analysis
GS
UKB
UKB_F
UKB_M
Cohort
0.0
0.1
0.2
0.3
Prop. of Trait Variance explained by GxSmoking

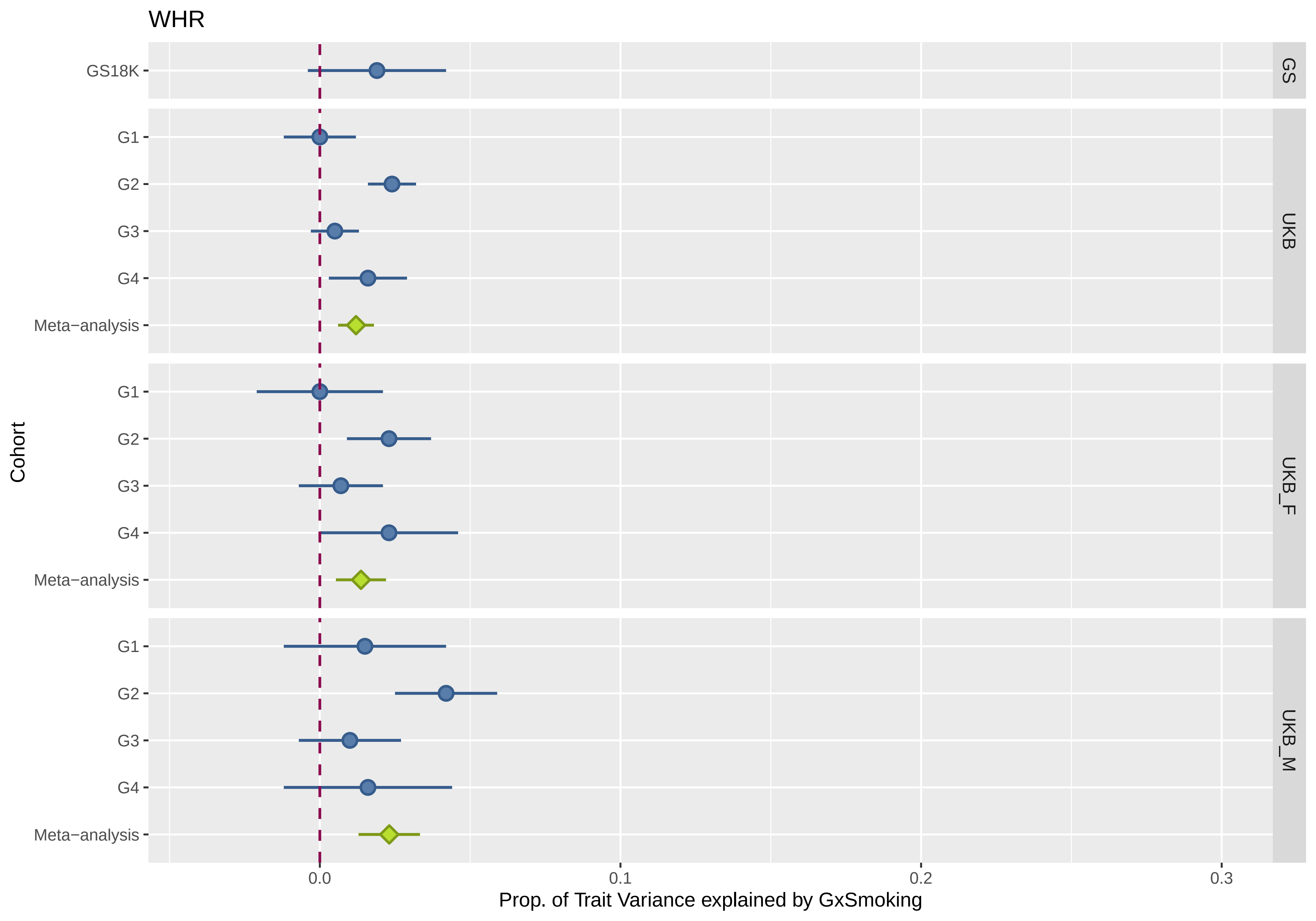

WHR
GS18K
G1
G2
G3
G4
Meta−analysis
G1
G2
G3
G4
Meta−analysis
G1
G2
G3
G4
Meta−analysis
GS
UKB
UKB_F
UKB_M
Cohort
0.0
0.1
0.2
0.3
Prop. of Trait Variance explained by GxSmoking

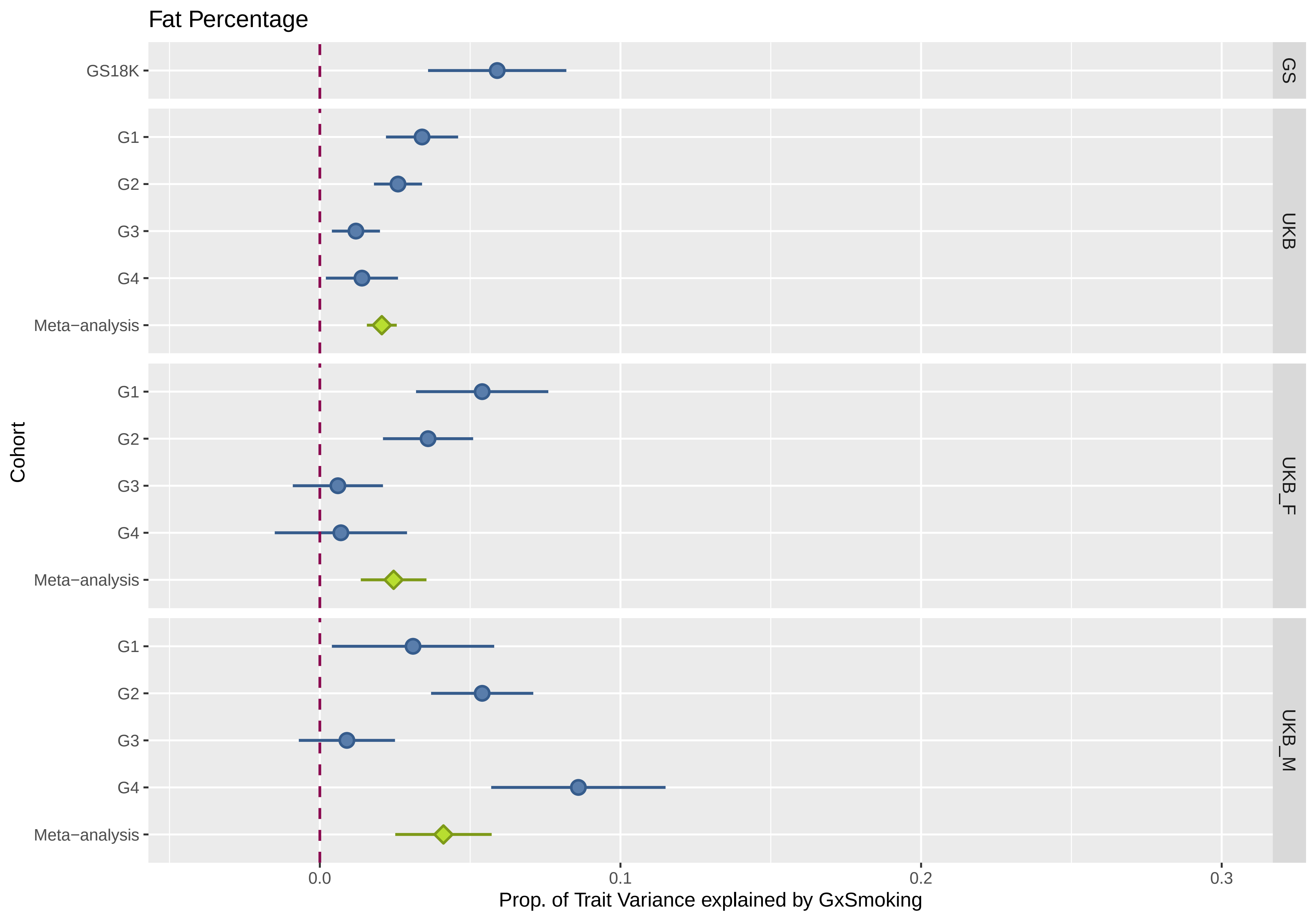
Fat Percentage
GS18K
G1
G2
G3
G4
Meta−analysis
G1
G2
G3
G4
Meta−analysis
G1
G2
G3
G4
Meta−analysis
GS
UKB
UKB_F
UKB_M
Cohort
0.0
0.1
0.2
0.3
Prop. of Trait Variance explained by GxSmoking
